# Enhanced atrial internal-external neural remodeling facilitates atrial fibrillation in the chronic obstructive sleep apnea model

**DOI:** 10.1371/journal.pone.0247308

**Published:** 2021-02-19

**Authors:** Jiasuoer Xiaokereti, Yan-Kai Guo, Zhen-Yu Dong, Mei Ma, Yan-Mei Lu, Yao-Dong Li, Xian-Hui Zhou, Ling Zhang, Bao-Peng Tang

**Affiliations:** 1 Cardiac Pacing and Electrophysiological Department, The First Affiliated Hospital of Xinjiang Medical University, Urumqi, Xinjiang, China; 2 Xinjiang Key Laboratory of Cardiac Electrophysiology and Cardiac Remodeling, The First Affiliated Hospital of Xinjiang Medical University, Urumqi, Xinjiang, China; 3 Department of Cardiology, The Fifth Affiliated Hospital of Xinjiang Medical University, Urumqi, Xinjiang, China; 4 Department of Teaching Management, The First Affiliated Hospital of Xinjiang Medical University, Urumqi, Xinjiang, China; Scuola Superiore Sant’Anna, ITALY

## Abstract

**Objective:**

Autonomic imbalance plays a crucial role in obstructive sleep apnea (OSA) associated atrial fibrillation (AF). Here, we investigated the potential neural mechanism of AF induced by OSA.

**Methods:**

Ten dogs were divided into control group (n = 5) and OSA group (n = 5). The chronic OSA model was established by repeat apnea-ventilation cycles for 4 hours a day for 12 weeks. During the process of model establishment, arterial blood gases, atrial effective refractory period (AERP), AF inducibility, normalized low-frequency power (LFnu), normalized high-frequency power (HFnu), and LFnu/ HFnu were evaluated at baseline, 4^th^ week, 8^th^ week, and 12^th^ week. Nerve activities of left stellate ganglion (LSG) and left vagal nerve(LVN) were recorded. Tyrosine hydroxylase(TH), choline acetyltransferase(CHAT), PGP9.5, nerve growth factor(NGF), and c-Fos were detected in the left atrium, LSG, and LVN by immunohistochemistry and western blot. Moreover, high-frequency stimulations of LSG and LVN were conducted to observe the AF inducibility.

**Results:**

Compared with the control group, the OSA group showed significantly enhanced neural activity of the LSG, increased AF inducibility, and shortened AERP. LFnu and LFnu/HFnu were markedly increased in the OSA group, while no significant difference in HFnu was observed. TH-positive and PGP9.5-positive nerve densities were significantly increased in the LSG and left atrium. Additionally, the protein levels of NGF, c-Fos, and PGP9.5 were upregulated both in the LSG and left atrium. AF inducibility was markedly increased under LSG stimulation without a stimulus threshold change in the OSA group.

**Conclusions:**

OSA significantly enhanced LSG and left atrial neural remodeling, and hyperactivity of LSG may accelerate left atrial neural remodeling to increase AF inducibility.

## Introduction

Atrial fibrillation (AF) is one of the most common clinical cardiac arrhythmias and is characterized by significant morbidity and mortality. Considerable evidences have shown that obstructive sleep apnea (OSA) is closely related to AF, such as increased recurrence rates after pulmonary vein isolation and the unsatisfactory efficacy of antiarrhythmic drug therapy [1–4]. Recently, several researchers have found a strong association between the atrial autonomic nervous system and OSA-induced AF [5–7].

The atrial autonomic nervous system consists of the internal autonomic system and external autonomic system, which are mainly derived from parasympathetic and sympathetic nerves [8, 9]. Yu et al. [6] revealed that OSA dramatically increased AF inducibility and left renal sympathetic activity, which could be inhibited by low-level tragus stimulation. Furthermore, Linz et al. [10] observed that negative tracheal pressure-induced atrial effective refractory period (AERP) shortening in OSA-related AF was alleviated by renal denervation. Additionally, metoprolol treatment reversed atrial remodeling and inhibited cardiac sympathetic nerve hyperactivity [11]. Moreover, Yang et al. [12] found that cardiac sympathetic denervation reduced AF susceptibility in a rat model of chronic intermittent hypoxia. The abovementioned animal studies showed sympathetic hyperactivity promoting AF inducibility in OSA. However, the roles of the external and atrial autonomic nervous system in OSA-related AF are still unclear. We hypothesized that the OSA increased AF inducibility partly by enhancing left stellate ganglion (LSG) activity and atrial neural remodeling.

## Material and methods

### Animal preparation

The experimental study protocol was reviewed and approved by the Institutional Animal Care and Use Committee of the First Affiliated Hospital of Xinjiang Medical University (Approval Number: IACUC201902-K04) and conducted by the principle of the Association for Assessment and Accreditation of Laboratory Care (AAALAC) and Basel Declaration. Ten adult male beagle dogs (weighing 18–22 kg, supplied by the Nanjing Yadong Experimental Animal Research Center) were included. Each dog was housed in a single block of kennel with a 12-h light and 12-h dark cycle, and allowed free access to food and water. Sodium pentobarbital (30 mg/kg) was injected intravenously to minimize animal suffering during the surgical procedure.

### The experimental model of chronic OSA

Ten adult male beagle dogs were randomized into two groups: the control and OSA groups. The chronic OSA model was established by 4 hours/day apnea-ventilation cycles for 12 weeks. Specific modeling method was based on a previous research protocol [11, 13]. In brief, the chronic OSA modeling process continued for 12 weeks for 4 hours a day, and apnea was mimicked by blocking the tracheal cannula for 1 min at the end of exhalation. All dogs from the OSA group were anesthetized via intramuscular injection with a mixture of Zoletil (0.1 mg/kg) (Virbac S.A. France) and xylazine (5 mg/kg) (Huamu Animal Health Care Products Co., Ltd., China), followed by sodium pentobarbital (50 to 80 mg/kg) as necessary. The apnea time was always set as 1 minute. In the first week, the ventilation time was set as 9 minutes, and the apnea-hypopnea index (AHI) was 6. Then, the ventilation time was reduced by 1 minute weekly until the fourth week. For the last eight weeks, the ventilation time was set as 5 minutes, and the AHI was 10. For the control group, we only gave intubation and anesthesia 4 hours a day without modeling.

### Arterial blood gas measurements

Arterial blood samples were drawn through femoral artery at preapnea and postapnea for blood gas analysis (PaO_2,_ PaCO_2_, and pH). Arterial blood gases were measured at baseline and at the end of 4^th^ week, 8^th^ week, and 12^th^ week to analyze the changes further.

### Atrial electrophysiology measurements

At baseline and every four weeks after the start of modeling, all dogs received programmed stimulation by LEAD-7000 (Jinjiang Electronic Science and Technology Inc., Chengdu, China) with a 10-pole electrode catheter that was placed in the high right atrium. The AERP was measured by eight S1-S2 programmed stimuli (initial S1-S1 = 330 ms) followed by a premature atrial stimulation (twofold threshold, pulse width of 0.5 ms). The S1-S2 interval was reduced by 5 ms each time until S2 failed to induce depolarization. The window of vulnerability (WOV, difference between the maximum and the minimum S1-S2 intervals) was used to quantify AF inducibility during AERP measurements by decreasing S1-S2 stimulation. AF inducibility was induced by burst pacing applied to the right atrium at 1200 bpm, fourfold threshold for 30 s. AF inducibility was measured ten times under ventilation and apnea at baseline, 4^th^ week, 8^th^ week, 12^th^ week, respectively. AF inducibility was counted as the percentage of successfully induced AF. AF was defined as irregular, rapid atrial beats >500 beats per minute (bpm) sustained for longer than 5 s [14].

### Cardiac ultrasound measurements

The cardiac echocardiographic examinations were evaluated by an experienced sonographer who was blinded to the study with a PHILIPS HD11XE imaging system (Philips Inc., Bothell, WA). The left atrial diameter and right atrial diameter were measured and recorded over at least three cardiac cycles. The left ventricular ejection fraction was measured and calculated by Simpson’s biplane method on apical-2 and 4 chamber views [15].

### Heart rate variability (HRV) measurements

Spectral power for HRV parameters were measured in 5-minute segment under nonanesthetized conditions at baseline, the 4^th^ week, the 8^th^ week, and the 12^th^ week by a data acquisition system (ML866/P; ADInstruments, Bella Vista). All data were analyzed using LabChart 8.0/proV7 software (ADInstruments, Australia). The HRV indexes were recorded as the frequency domain index, including normalized low-frequency power (LFnu, 0.04–0.15 Hz, reactive sympathetic nerve activity), normalized high-frequency power (HFnu, 0.15–0.40 Hz, reactive parasympathetic nerve activity), and LFnu/HFnu (reactive sympathetic and parasympathetic nerve balance) [16].

### Neural activity recording

After 12 weeks of modeling, the left vagal nerve (LVN) and LSG were continuously recorded for 3 hours through cervicothoracic junction. Specific methods of nerve separation and recording were previously reported by our team [17, 18]. The nerve signals were amplified with a PowerLab DP-301 differential amplifier (ML866/P; ADInstruments, Bella Vista), and bandpass filters were set as 160 Hz (low-pass) to 200 Hz (high-pass) for the LVN and 100 Hz (low-pass) to 1000 Hz (high-pass) for the LSG. Neural activity was manually calculated and analyzed by recording frequency and amplitude with a signal-to-noise ratio >3:1 [19, 20].

### AF inducibility measurements by LSG and LVN stimulation

At the end of nerve recording, a hook stimulating electrode was used to stimulate the LSG with a Grass S88 stimulator (Astro-Med Inc, USA). The initial stimulator parameters were set as 20 Hz, 0.1-ms pulse width, and 1 V. To detect stimulus threshold of LSG, the stimulation voltage was gradually increased by 1 V until the heart rate or blood pressure rose to 120% of baseline levels [21]. AF inducibility was induced by LSG stimulation at 20 Hz, the twofold threshold for 40 s. LSG stimulation repeated five times for each dog after heart rate and blood pressure returned to prestimulus baseline levels. The simulation was stopped when AF was successfully induced. AF inducibility was counted as the percentage of successfully recorded AF.LVN stimulation was performed after LSG was evaluated. The precise method was the same as mentioned above. In contrast, the initial stimulator parameters for the LVN were set as 20 Hz, 0.1 ms width, and 0.5 V. Additionally, the stimulation voltage was gradually increased by 0.5 V, and the stimulus threshold of the LVN was defined as the current required to produce a decline of 20% heart rate.

## Histology analysis

Finally, the left atrial, LSG, and LVN tissues of dogs were obtained, immediately transferred to 4% paraformaldehyde, embedded in paraffin, and sliced into 5-μm-thick cross-sections. Hematoxylin-eosin (HE) staining was used to observe the morphology of the atrial. Masson trichrome staining was used to evaluate atrial fibrosis. These histological changes were observed by microscopy (Zeiss, Germany). Image-Pro Plus 6.0 software (Media Cybernetics, USA) was used to calculate the area of the interstitial fiber.

The streptavidin-peroxidase immunohistochemical method was used to detect the expression levels of TH (tyrosine hydroxylase, 1:100; Novus, Colorado, USA), CHAT (choline acetyltransferase, 1:200; Bioss, Beijing, China), PGP9.5 (1:100; Novus, Colorado, USA), connexin-40 (Cx40;1:200, Abcam, Cambridge, UK), and connexin-43 (Cx43,1:600; Abcam, Cambridge, UK). Immunohistochemical staining was observed and imaged under a microscope (Leica, Wetzlar, Germany), and analyzed by Image-Pro Plus 6.0 software. The nerve fiber density was counted in 5 serial sections from each dog at 200× magnification and is shown as the ratio of positive nerve area to total area (μm^2^/mm^2^). Additionally, integrated optical density (IOD) sum was used for quantitative analysis of Cx40 and Cx43.

### Western blot analysis

The protein expression of nerve growth factor (NGF), c-Fos, PGP9.5, TH, and CHAT was detected by western blot based on previously described methods [22]. Briefly, 40 μg of protein was separated by SDS-PAGE and transferred to polyvinylidene difluoride (PVDF) membranes. The membranes were blocked with 5% nonfat milk for 1 h at room temperature and incubated with primary antibodies overnight at 4°C. Then, the membranes were washed and incubated with a secondary antibody conjugated with horseradish peroxidase for 2 h. Finally, the protein bands were visualized using enhanced chemiluminescence (Thermo Scientific, MA, USA) in a darkroom. Image-Pro Plus 6.0 software was used to analyze the protein band density. The primary antibodies were as follows: anti-NGF (1:1000; Novus Biologicals, California, USA), anti-c-Fos (1:1000; LSBio, Seattle, USA), anti-PGP9.5(1:1000; Novus Biologicals, California, USA), anti-TH (1:300; Novus Biologicals, California, USA), and anti-CHAT (1:1000; Bioss, Beijing, China). Relative protein expression levels were normalized to the GAPDH expression level (1:1000; Abcam, Cambridge, UK).

### mRNA analysis

Quantitative real-time polymerase chain reaction (qRT-PCR) was performed to quantify the mRNA expression levels of NGF, c-Fos, Kir3.1, Nav1.5, Cav1.2, Cx40, and Cx43. According to the manufacturer’s instructions, total RNA was extracted with TRIzol reagent (Takara, Tokyo, Japan) and reverse-transcribed to cDNA using a PCR kit (Takara, Tokyo, Japan). The amplification reaction was repeated for 55 amplification cycles of 10 seconds at 95°C, 20 seconds at 58°C, and 10 seconds at 72°C. The specificity of amplification was confirmed by melting curve analysis. GAPDH was used as an internal control to correct for variability between samples, and all data were analyzed using the comparative CT method (2^-ΔΔCt^). The primer sequences and amplicon sizes of the related genes are listed in **Table 1.**

**Table 1:** Primer sequences for RT-qPCR.

Gene Name	Accession No.	Primer Sequence	Amplicon Size(bp)
c-FOS	XM_547914.5	F: 5’-CTTCATCCCGACGGTGACT-3’	175
		R: 5’-TCCCGTCATGGTCTTCACAA-3’	
NGF	NM_001194950.1	F: 5’-CAATAGCTGCCAGGGTGACA-3’	179
		R: 5’-GTGAGTCCTGTTGACGGAGG-3’	
GJA5(Cx40)	NM_001017442	F: 5’-ATGGCTGAGTGTCAGCTTCC-3’	144
		R: 5’-GTTCTGCTGGGAAGCCATCT-3’	
GJA1(Cx43)	NM_001002951.3	F: 5’-AGAGAACCCTGCCCTCATCA-3’	174
		R: 5’-AGGATCGCTCTTGCCTTTCA-3’	
KCNJ3(Kir3.1)	XM_545477.5	F: 5’-AGCGAAGCATGCAAACTGAA-3’	195
		R: 5’-CAAACGTCGCGTGGAATTGG-3’	
SCN5A(Nav1.5)	NM_001002994.1	F: 5’-CGTGGGAGCATCTTCACCTT-3’	189
		R: 5’-TTTACCATTGAGGGCGGGAG-3’	
CACNA1C(Cav1.2)	XM_014108292.1	F: 5’-ACCAGGCATCTTCGTTGGAG-3’	116
		R: 5’-AGATGCGGGAGTTCTCCTCT-3’	
GAPDH	NM_001003142.2	F: 5’-TAGTGAAGCAGGCATCGGAG-3’	102
		R: 5’-AGGTGGAAGAGTGGGTGTCA-3’	

Abbreviations: RT-qPCR: Quantitative Real-time PCR; NGF: Nerve growth factor; Cx40: Connexin 40; Cx43: Connexin 43.

### Statistical analysis

Comparisons of data between 2 groups were performed by using unpaired t-tests when the data conformed to a normal distribution, or by the Mann–Whitney U test. The Shapiro-Wilk normality test was used to test whether the variables complied with a normal distribution. A paired t-test was used to evaluate blood gas values before and after apnea. Tow-way repeated measures ANOVA followed by the Sidak and Tukey *post hoc* test was used for the comparisons of repeated measures quantitative variables. Fisher’s exact test was performed to compare proportions. All data were presented as the mean±SD except for the stimulus thresholds of LSG and LVN, which were reported as the median with 25th to 75th interquartile ranges. GraphPad Prism 8.0.1 (GraphPad Software, Inc., San Diego, California) software was used for statistical analysis and plotting graphics. A two-sided *P* < 0.05 was considered statistically significant.

## Result

### Arterial blood gases analysis

**Fig 1:**
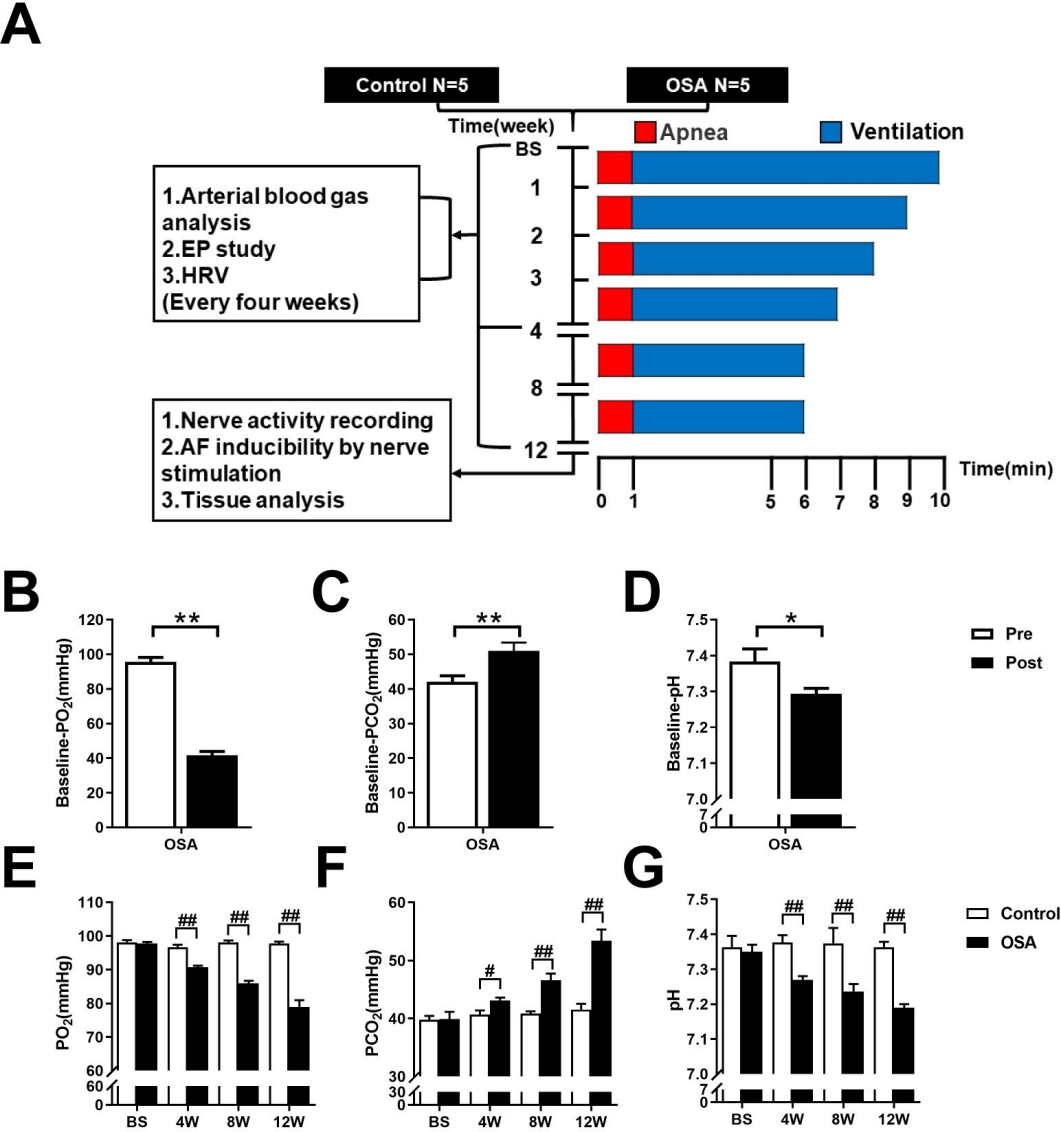
Flow chart of the design and analysis of arterial blood gas changes. (A) Flow chart of the study design. (B-D) Arterial blood gas analysis before and after 1 minute of apnea at baseline. (E-G) Arterial blood gas analysis between control and OSA groups at the end of the 4^th^, the 8^th^, and the 12^th^ week. Abbreviations: EP: electrical physiology; HRV: heart rate variability; Pre: preapnea; Post: postapnea. *P <0.05, **P <0.01 compared to preapnea; ^#^*P* <0.05, ^##^*P* <0.01 compared to control group.

To validate the OSA model, we collected arterial blood samples from the femoral artery before and after 1 minute of apnea at baseline. The pH, PO_2_, and PCO_2_ were measured. As compared to preapnea, the pH and PO_2_ were decreased (7.38 ± 0.04 *vs*. 7.29 ± 0.02, *P* = 0.0319; 95.62 ± 2.51 *vs*. 41.66 ± 2.15 mmHg, *P* = 0.0001), whereas the PCO_2_ was significantly increased (42.05 ± 1.72 *vs*. 50.99 ± 2.38 mmHg, *P* = 0.0062) after 1 minute of apnea (Fig 1B–1D).

Moreover, we performed arterial blood gas analysis at baseline and every four weeks. Compared with those in the control group, PO_2_ and pH were markedly decreased in the OSA group beginning at the second time point (4^th^ week) (all *P* value < 0.01), whereas PCO_2_ was significantly increased (all *P* value < 0.05) (Fig 1E–1G and S1 File).

### Related atrial electrophysiology changes

**Fig 2:**
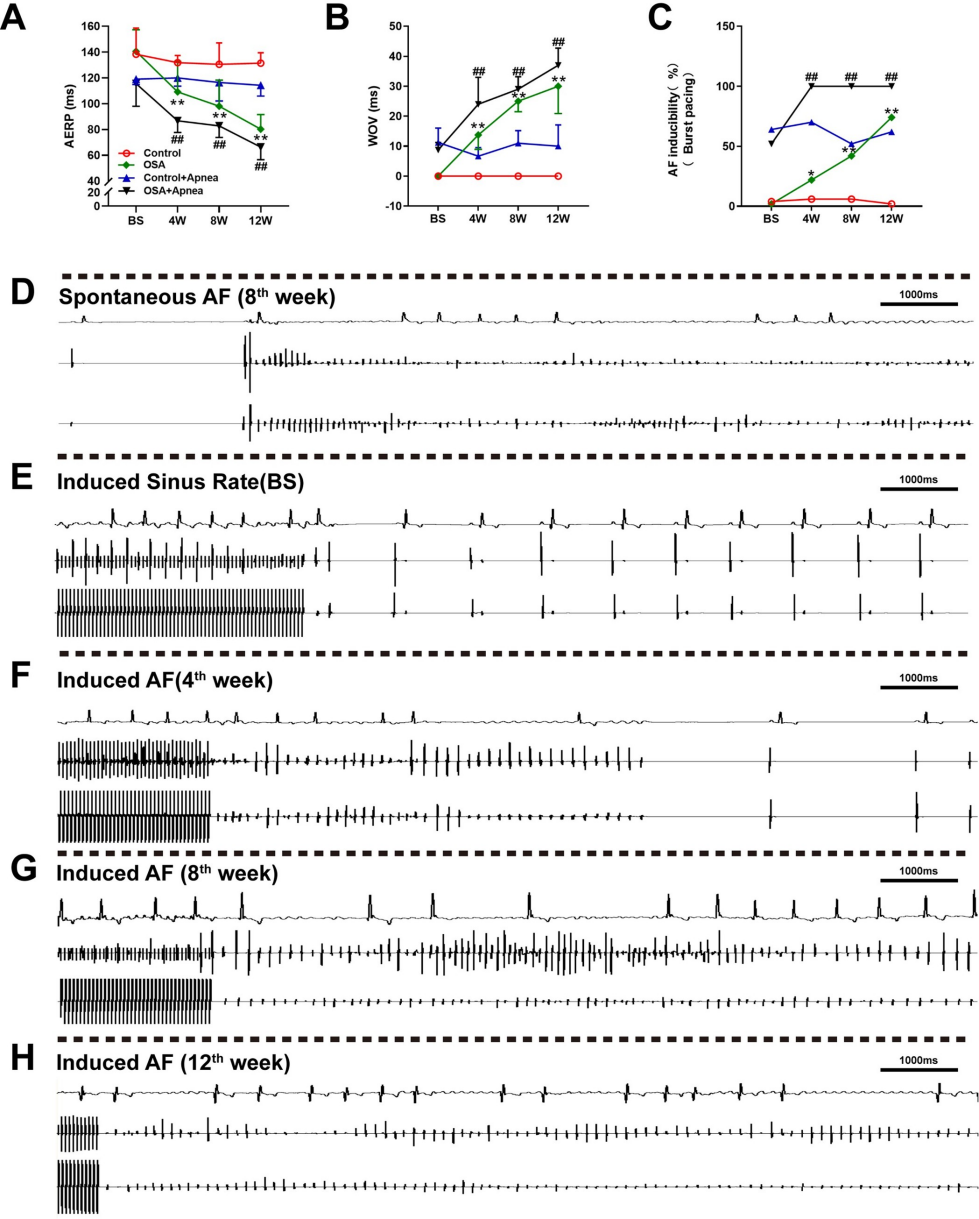
Related electrophysiology changes. (A) Comparison of the AERP between the two groups. (B) Comparison of the WOV between the two groups. (C) Comparison of AF inducibility between the two groups under burst pacing. (D) Spontaneous AF event observed in the OSA group. (E) Sinus rhythm indued by burst pacing in the OSA group at baseline. (F-H) AF indued by burst pacing in the OSA group at different time points. *P <0.05, **P <0.01 compared to control group under ventilation condition; ^#^*P* <0.05, ^##^*P* <0.01 compared to control under apnea condition.

All dogs received intracardiac electrophysiological examination weekly, and all dogs survived during the research period. No significant differences in either the AERP or WOV between the two groups were observed at baseline (all *P* values> 0.05). Compared with the control group, OSA shortened the AERP after 4^th^ week (all *P* value < 0.01) (Fig 2A). For example, the AERPs at the 4^th^ week were 131.81 ± 5.45 *vs*. 109.16 ± 23.47ms (*P* = 0.0051) and 120.00 ± 6.45 *vs*. 86.67 ± 9.01ms (*P* < 0.0001) under apnea and ventilation. In contrast, the WOVs were significantly prolonged after 4^th^ week compared with the control group (all *P* value < 0.01) (Fig 2B). After the first four weeks, AF inducibility was markedly increased under the two conditions compared with that in the control group (ventilation: 6% *vs*. 22%, apnea: 70% *vs*. 100%, all *P* value < 0.05) (Fig 2C and S1 File). Meanwhile, during the electrophysiological examination, a certain number of spontaneous AF events were observed in the OSA group (Fig 2D). Typical results of burst pacing were presented in Fig 2E–2H.

### HRV changes

**Fig 3:**
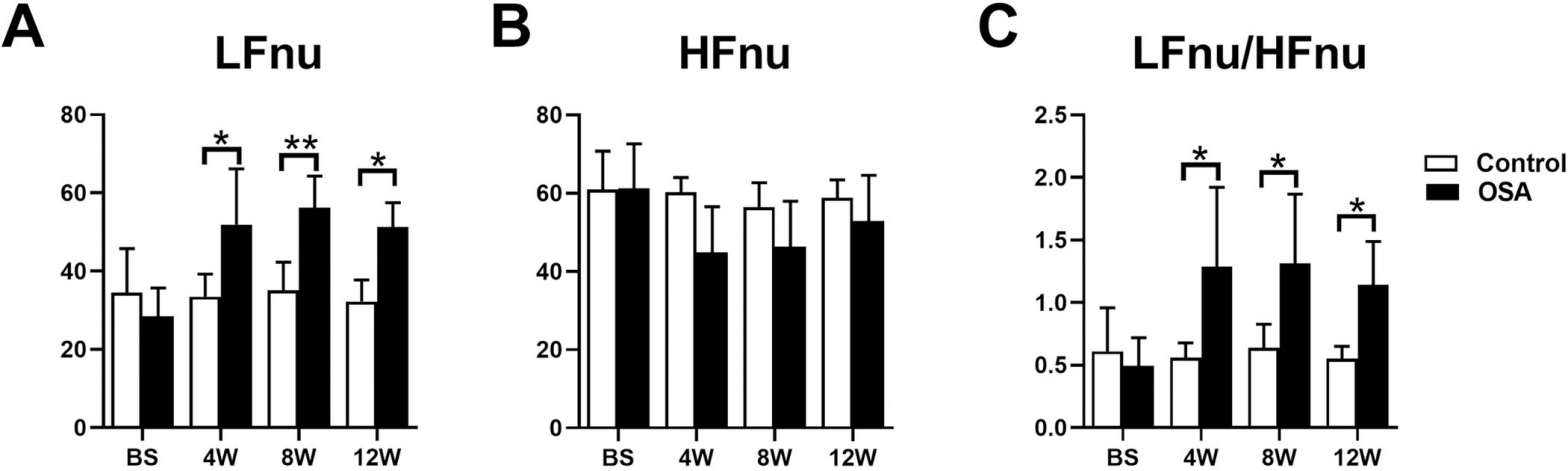
Comparison of HRV between the two groups. *P <0.05, **P <0.01 compared to control group.

HRV indicators, including LFnu, HFnu, and LFnu/HFnu were recorded in 5-minute periods. No significant differences in LFnu, HFnu, or LFnu/HFnu were observed between the control and OSA group at baseline (all *P* value >0.05). Compared with those in the control group, LFnu and LFnu/HFnu were markedly increased in the OSA group at the 4^th^ week (LFnu: 33.48 ± 5.73 *vs*. 52.00 ± 14.16, *P* = 0.0148; LFnu/HFnu: 0.56 ± 0.12 *vs*. 1.29 ± 0.64, *P* = 0.0141), 8^th^ week (LFnu: 35.12 ± 7.15 *vs*. 56.23 ± 8.07, *P* = 0.0054; LFnu/HFnu: 0.64 ± 0.19 *vs*. 1.31 ± 0.55, *P* = 0.0187), and 12^th^ week (LFnu: 32.25 ± 5.54 *vs*. 51.28 ± 6.24, *P* = 0.0121; LFnu/HFnu: 0.55 ± 0.10 *vs*. 1.14 ± 0.35, *P* = 0.03), while no significant differences in HFnu were observed between the two groups (all *P* value >0.05) (Fig 3 and S1 File).

### Atrial nerve distribution and remodeling

**Fig 4:**
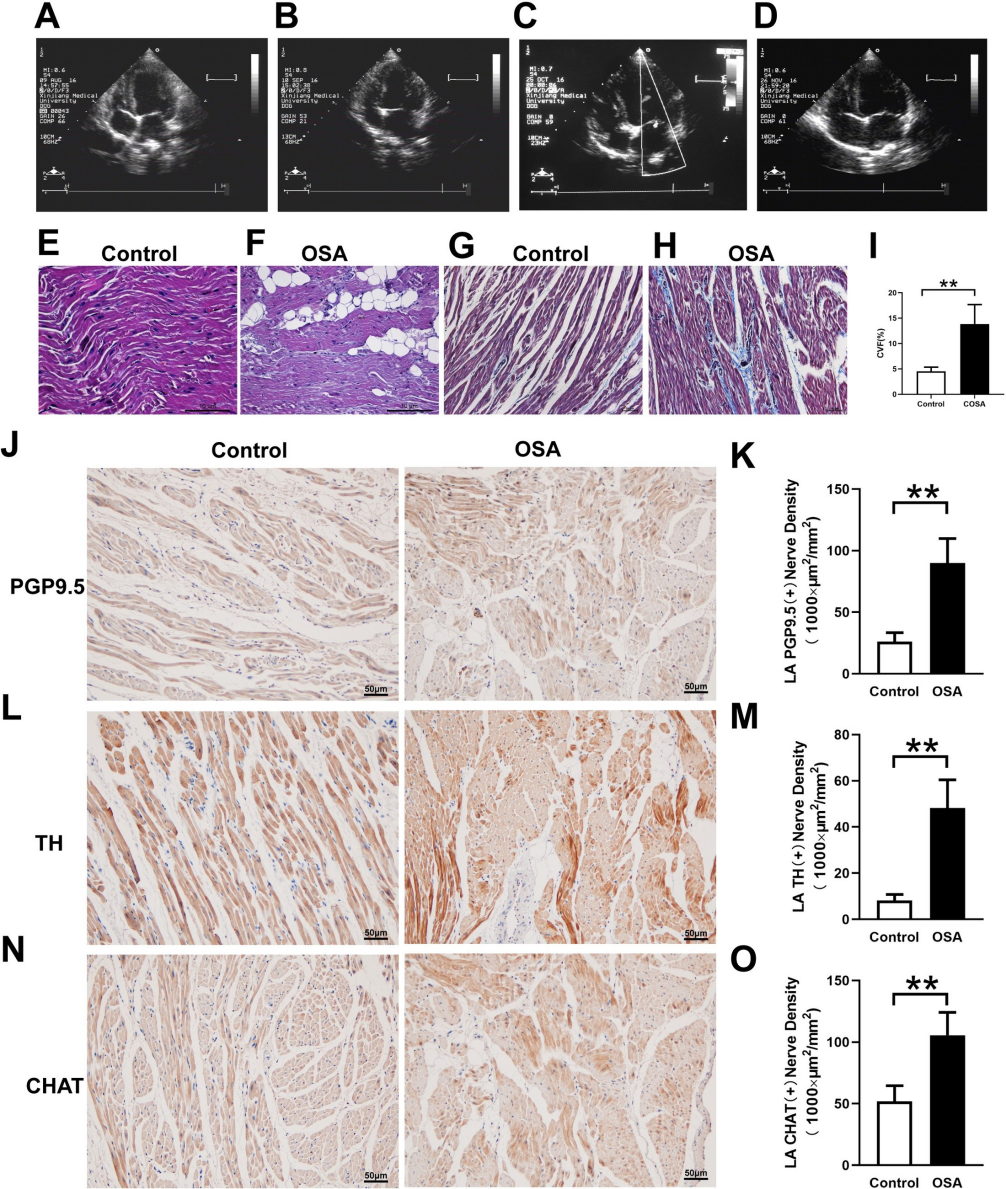
Atrial structural changes and neural remodeling in the atrial tissues. (A-D) Cardiac ultrasound results show that the left atrial diameter was gradually increased from 8^th^ week (A: Baseline. B: 4^th^ week. C: 8^th^ week. D: 12^th^ week). (E, F) HE staining of the left atrium. (G-I) Masson trichrome staining of the left atrium and semiquantitative evaluations of atrial fibrosis. (J-O)Analysis of the immunohistochemical expression of TH, CHAT, and PGP9.5 in the left atrium. Abbreviations: CVF: collagen volume fraction; TH: tyrosine hydroxylase; CHAT: choline acetyltransferase.*P <0.05, **P <0.01 compared to control group.

**Fig 5:**
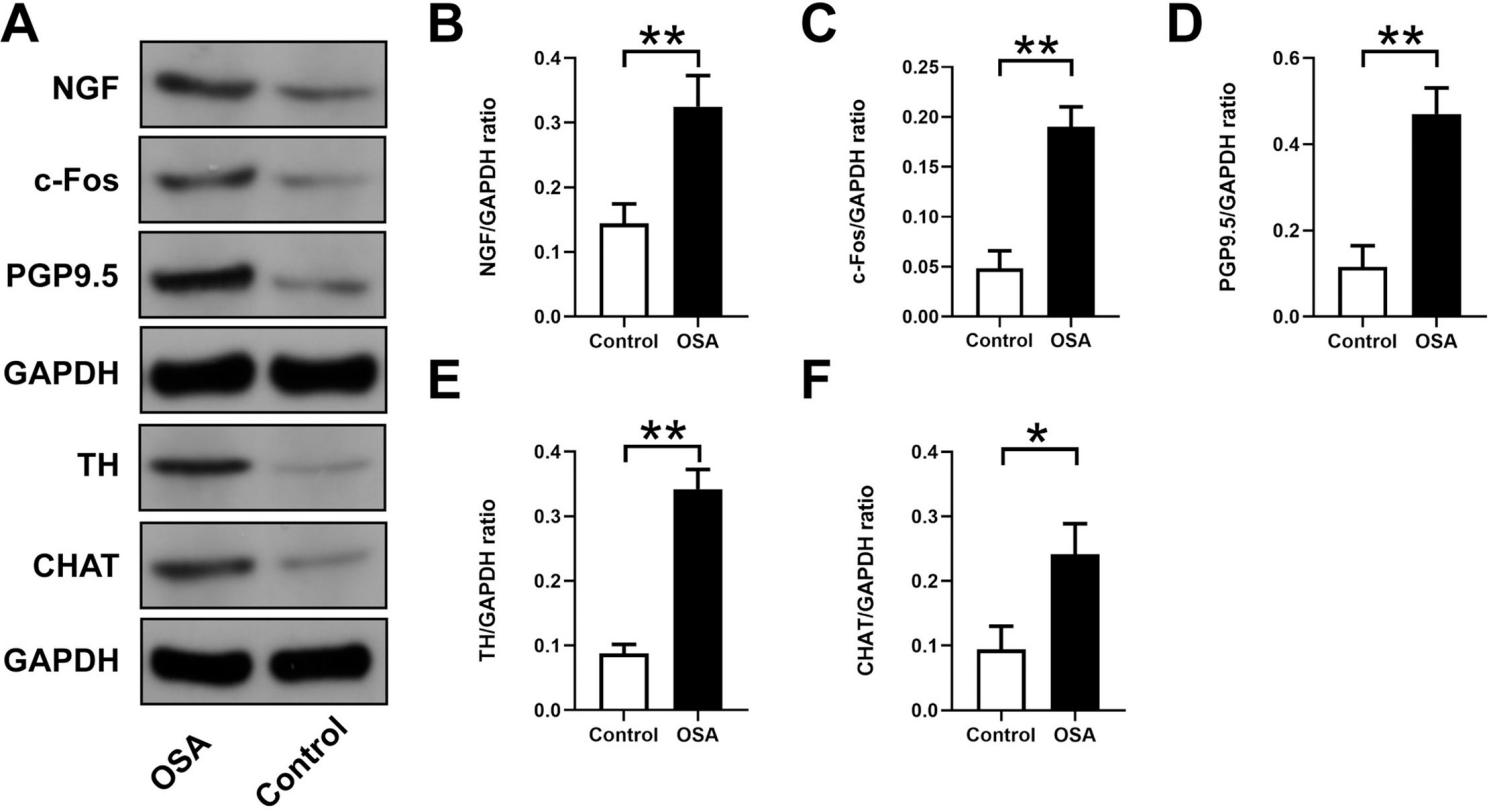
Expression levels of NGF, c-Fos, PGP9.5, TH, and CHAT in atrial tissues. (A) Western blotting results for NGF, c-Fos, PGP9.5, TH, and CHAT in the two groups. Relative protein expression of NGF (B), c-Fos (C), PGP9.5 (D), TH (E), CHAT (F) are shown and normalized to GAPDH. Abbreviations: NGF: nerve growth factor; TH: tyrosine hydroxylase; CHAT: choline acetyltransferase.*P <0.05, **P <0.01 compared to control group.

**Fig 6:**
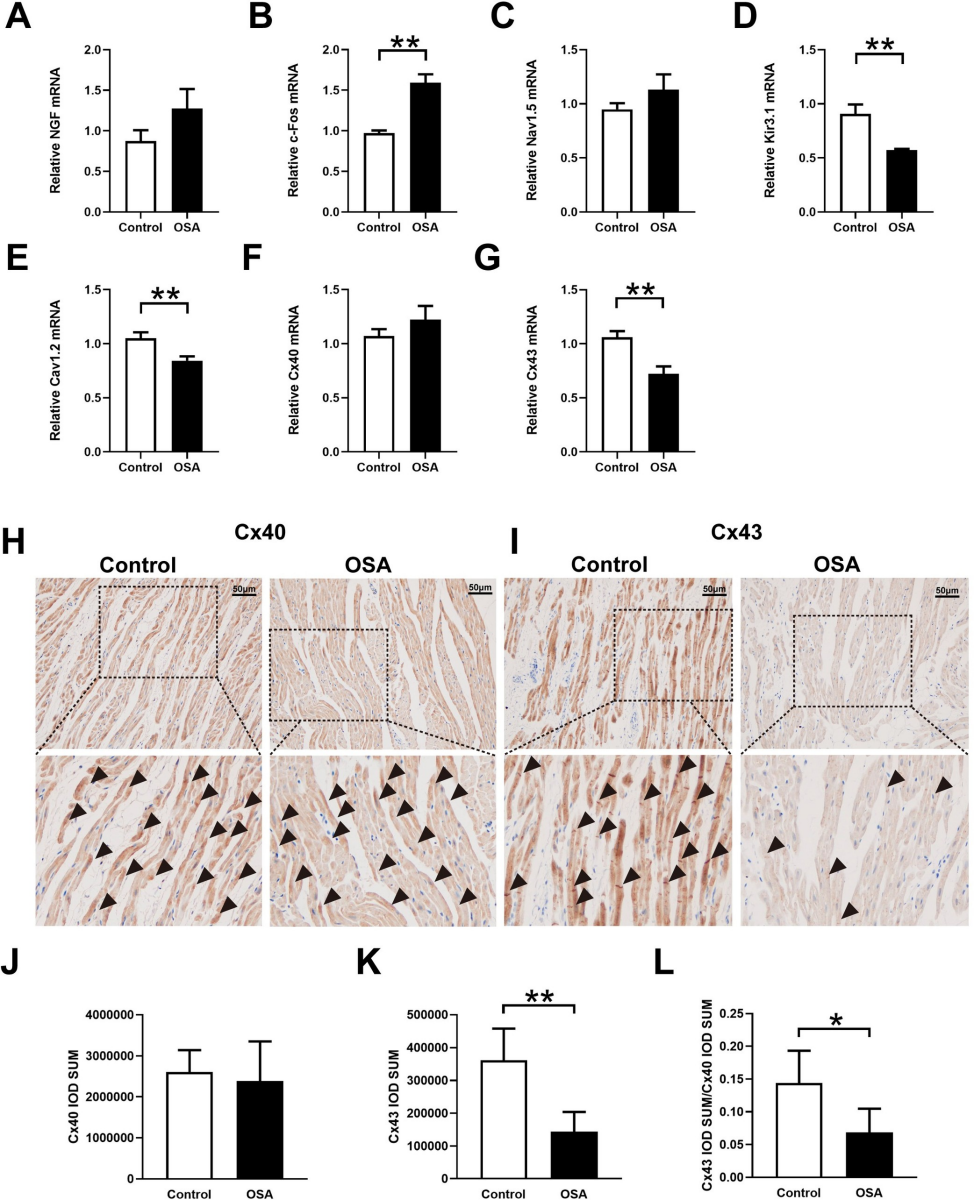
Neural remodeling and ion channels related to expression analysis. (A-G) Expression levels of NGF (A), c-Fos (B), Nav1.5 (C), Kir3.1 (D), Cav1.2 (E), Cx40 (F), Cx43 (G). H, I, Representative immunohistochemistry of Cx40 and Cx43 in the left atrium. J-L, Quantitative analysis of the immunohistochemical expression of Cx40 and Cx43. Abbreviations: NGF: nerve growth factor; Cx40: connexin-40; Cx43: connexin-43.*P <0.05, **P <0.01 compared to control group.

Fig 4J–4O showed immunohistochemical staining in the control group and OSA group. In contrast to those in the OSA group, PGP9.5-positive (26.04 ± 7.41 *vs*. 90.00 ± 19.78×1000 μm^2^/mm^2^, *P* = 0.0001) (Fig 4J and 4K), TH-positive (8.18 ± 2.57 *vs*. 48.21 ± 12.22×1000 μm^2^/mm^2^, *P* <0.0001) (Fig 4L and 4M), and CHAT-positive (51.90 ± 12.70*vs*. 105.6 ± 18.57 ×1000 μm^2^/mm^2^, *P* = 0.0007) (Fig 4N and 4O) nerve densities were significantly increased. Furthermore, the Western blot results of PGP9.5 (0.12 ± 0.05 *vs*. 0.47 ± 0.06, *P* = 0.0014), TH (0.09 ± 0.01 *vs*. 0.34 ± 0.03, *P* = 0.0002), and CHAT (0.09 ± 0.04 *vs*. 0.24 ± 0.05, *P* = 0.0125) were significantly upregulated in the left atrium compared with the control group (Fig 5). Compared with the control group the mRNA expression of c-Fos was significantly increased (0.97 ± 0.03 *vs*. 1.60 ± 0.10, *P* = 0.0006), while no significant difference was observed in NGF (0.88 ± 0.13 *vs*. 1.27 ± 0.24, *P* = 0.0657) (Fig 6A and 6B). Moreover, protein expressions of NGF (0.14 ± 0.03 *vs*. 0.32 ± 0.05, *P* = 0.0053) and c-Fos (0.05 ± 0.01 *vs*. 0.19 ± 0.02, *P* = 0.008) were significantly upregulated in the left atrium compared with the control group (Fig 5B and 5C).

Cardiac ultrasound results showed that the left atrial diameter was gradually increased from week eight and did not impact the right atrial diameter and left ventricular ejection fraction (Fig 4A–4D and S1 File). The myocardial cells in the OSA group showed irregular shape, disordered arrangement, myocardial steatosis, inflammatory cell infiltration, and myocardial fibrosis (Fig 4E and 4F). Compared with the control group, the left atrium from chronic OSA dogs showed significantly higher levels of fibrosis (4.52 ± 0.83%vs. 13.82 ± 3.85%, P = 0.0007) (Fig 4E and 4F). Moreover, ion channels related to mRNA expression results were shown in Fig 6C–6E. Compared with that in the control group, expressions of Cav1.2 (1.05 ± 0.06 *vs*. 0.84 ± 0.04, *P* = 0.0063) and Kir3.1 (0.91 ± 0.09 *vs*. 0.57 ± 0.11, *P* = 0.0026) were significantly decreased, while the expression of Nav1.5 (0.95 ± 0.06 *vs*. 1.13 ± 0.14, *P* = 0.9493) was no statistical difference between two groups (Fig 6C–6E).

Previous researches have confirmed that the connexins are related to sympathetic nervous system-triggered AF [23–25]. Hence, we further determined the differential expressions of Cx40 and Cx43 in the left atrium by qRT-PCR and immunohistochemical staining. Compared with the control group the mRNA expression of Cx43 was significantly downregulated (1.06 ± 0.05 *vs*. 0.72 ± 0.09, *P* = 0.0027), and no significant difference was observed in Cx40 (1.07 ± 0.06 *vs*. 1.22 ± 0.13, *P* = 0.1415) (Fig 6F and 6G). Besides, compared with the control group, the expression of Cx43 in immunohistochemical staining was significantly decreased (361487 ± 96164 *vs*. 143746 ± 59677, *P* = 0.0026). There was no statistical difference of CX40 between the two groups (2607331 ± 530303 *vs*. 2386480 ± 964548, *P* = 0.6656) (Fig 6H–6K). Further analysis showed a significant decrease in Cx43/Cx40 ratio in the chronic OSA group (0.14 ± 0.04 *vs*. 0.069 ± 0.03, *P* = 0.0241) (Fig 6L).

### Neural activity changes

**Fig 7:**
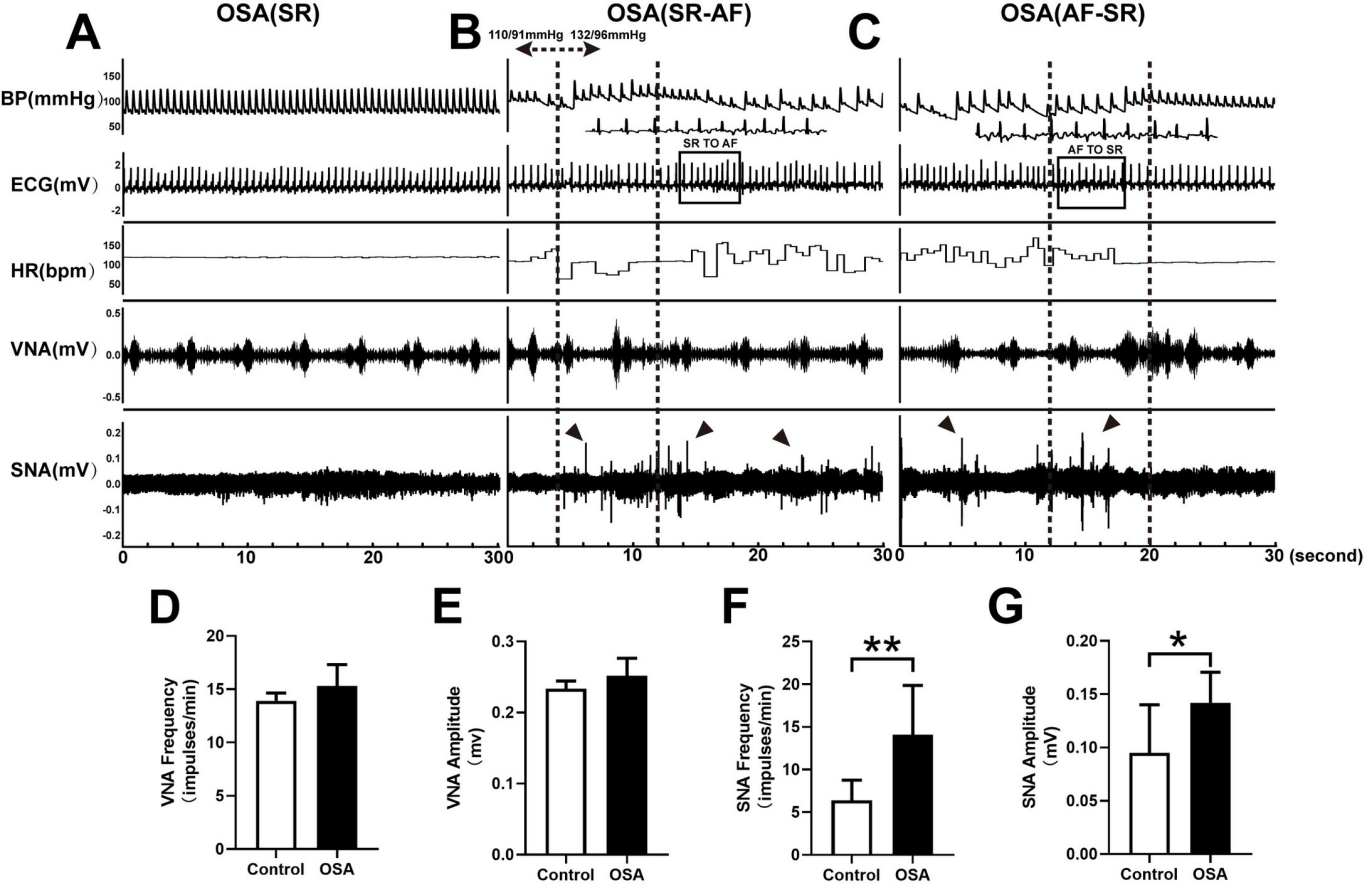
Recording and analysis of SNA, VNA in two groups. (A) In the OSA group, simultaneous recordings of SNA, VNA, ECG, HR, and BP in SR. (B)SR to AF. (C) AF to SR. (D) Nerve discharge frequency of the LVN. (E) Nerve discharge amplitude of the LVN. (F) Nerve discharge frequency of the LSG. (G) Nerve discharge amplitude of the LSG. Arrows: sympathetic discharge; Abbreviations: AF: atrial fibrillation; SR: sinus rhythm; ECG: electrocardiography; VNA: vagal activity; SNA: sympathetic activity; HR: heart rate; BP: blood pressure; LSG: left stellate ganglion; LVN: left vagal nerve.*P <0.05, **P <0.01 compared to control group.

Fig 7A–7C demonstrated representative examples of LVN and LSG neural activity in the OSA group after 12 weeks. Spontaneous AF was observed in the OSA group, with apparent nerve discharges of the LSG and increased blood pressure (Fig 7B). Fig 7C showed that nerve discharges of the LSG ended with the termination of spontaneous AF, and irregular discharges of the LVN were observed. Compared with the control group, the OSA group showed significantly enhanced nerve discharge frequency (6.38 ± 2.39 *vs*.14.10 ± 5.76 impulses/min, *P* = 0.0027) and amplitude (0.09 ± 0.045 *vs*. 0.14 ± 0.02 mV, *P* = 0.0158) of the LSG (Fig 7F and 7G). However, no significant differences were observed between the control and OSA group in the LVN (both *P-*value > 0.05) (Fig 7D and 7E).

### Nerve distribution in the LSG and LVN

**Fig 8:**
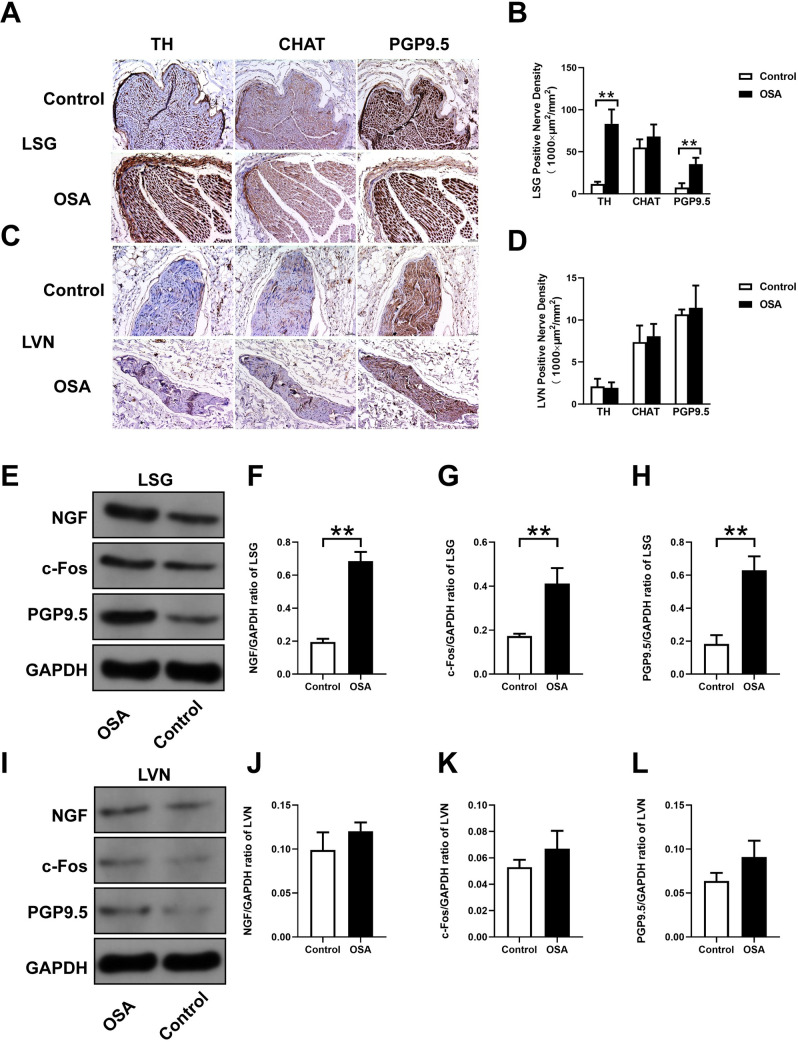
Nerve distribution in the LSG and LVN. (A, C) Representative immunohistochemical staining of TH, CHAT, and PGP9.5 in the LSG and LVN. (B, D) Quantification of TH, CHAT expression, and PGP9.5 in the LSG and LVN. (E, I) Western blotting results for NGF, c-Fos, PGP9.5 in the LSG and LVN. Relative protein expression of NGF (F, J) c-Fos (G, K), PGP9.5 (H, L) are shown and normalized to GAPDH. Abbreviations: LSG: left stellate ganglion; LVN: left vagal nerve; NGF: nerve growth factor; TH: tyrosine hydroxylase; CHAT: choline acetyltransferase. *P <0.05, **P <0.01 compared to control group.

Fig 8 showed immunohistochemistry and Western blot results in the LSG and LVN, related statistical results of TH, CHAT, PGP9.5, NGF, and c-Fos were shown as well. Compared with the control group, the LSG had remarkably higher TH-positive (11.96 ± 2.47 *vs*. 83.25 ± 17.06 ×1000 μm^2^/mm^2^, *P* < 0.0001) and PGP9.5-positive (7.59 ± 5.15 *vs*. 35.27 ± 7.66 ×1000 μm^2^/mm^2^, *P* < 0.0002) nerve densities, while there was no significant difference of CHAT-positive nerve density (55.02 ± 9.75 *vs*. 68.16 ± 14.33×1000 μm^2^/mm^2^, *P* = 0.1286) (Fig 8A and 8B). Additionally, in the LVN, CHAT-positive (7.38 ± 1.97 *vs*. 8.08 ± 1.46×1000 μm^2^/mm^2^, *P* = 0.5387), TH-positive (2.10 ± 0.91 *vs*.1.94 ± 0.65×1000 μm^2^/mm^2^, *P* = 0.7497), and PGP9.5-positive (10.67 ± 0.57 *vs*. 11.44 ± 2.65×1000 μm^2^/mm^2^, *P* = 0.5390) nerve densities had no significant changes in comparison to the control group (Fig 8C and 8D). Meanwhile, the protein expressions of NGF (0.19 ± 0.02 *vs*. 0.69 ± 0.05, *P* = 0.0001), c-Fos (0.17 ± 0.01 *vs*. 0.41 ± 0.07, *P* = 0.0043), and PGP9.5 (0.18 ± 0.05 *vs*. 0.63 ± 0.08, *P* = 0.0015) were significantly upregulated in the OSA group compared with the control group in the LSG (Fig 8E–8H), no significant changes were observed in the LVN (all *P* value >0.05) (Fig 8I–8L).

### LSG and LVN stimulation effects on OSA induced AF

**Fig 9:**
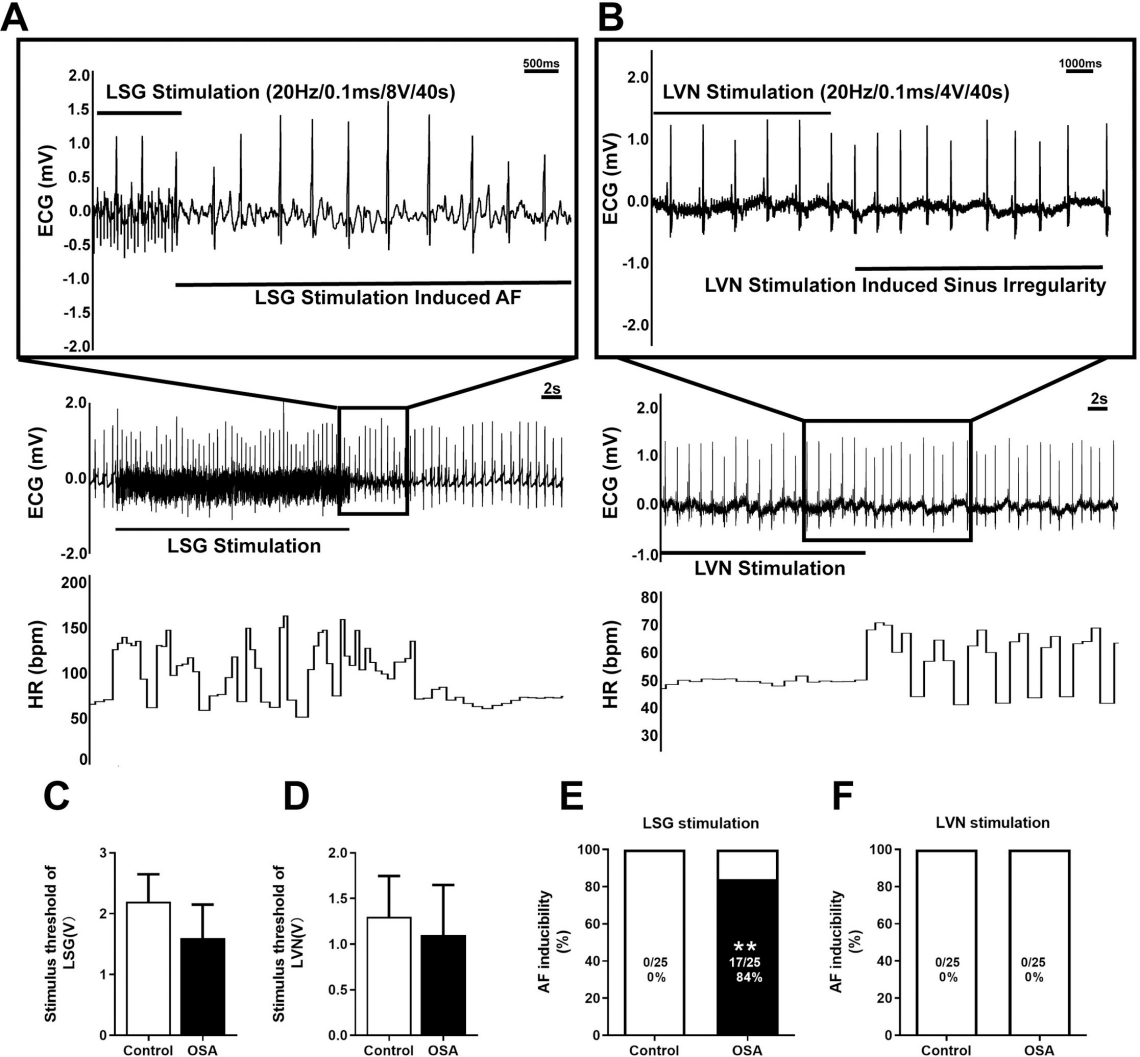
AF inducibility by LSG and LVN stimulation. (A) An example of induced AF in the OSA group under LSG stimulation. (B) An example of induced sinus arrhythmia in the OSA group under LVN stimulation. (C, D) Comparison of stimulus threshold of LSG and LVN. (E, F) Comparison of the AF inducibility under LSG and LVN stimulation. Abbreviations: LSG: left stellate ganglion; LVN: left vagal nerve.*P <0.05, **P <0.01 compared to control group.

Firstly, the LSG and LVN stimulus thresholds were carefully determined. Compared with the control group, the stimulus threshold of LSG decreased in the OSA group, but the difference was insignificant between the two groups. (control: 2 [0.5], OSA: 2 [1], *P* = 0.2778) (Fig 9C). Meanwhile, the stimulus threshold of LVN had no significant difference either (control: 1 [0.75], OSA:1 [0.75], *P* = 0.6429) (Fig 9D). Fig 9A and 9B demonstrated representative examples of LSG and LVN stimulation in the OSA group after 12 weeks. Moreover, the AF inducibility was markedly increased under the LSG stimulation compared with the control group (0% *vs*. 84%, *P* <0.0001) (Fig 9E). Simultaneously, no AF was induced in two groups when stimulated the LVN (Fig 9F).

## Discussion

### Major findings

The present study demonstrated that OSA significantly enhanced LSG activity and augmented neural remodeling in the atrium, leading to increased AF inducibility. Several lines of evidences support this conclusion. First, OSA decreased the AERP and increased the WOV over time, which indicated the instability of atrial electrophysiology. Second, the protein levels of TH, NGF, and c-Fos were upregulated in the LSG and left atrium in the OSA group. Third, LFnu and LFnu/HFnu increased from the 4^th^ week until the end of the experiment, which were consistent with the electrophysiological changes.

### OSA increased AF inducibility and reduced AERP over time

Zhao et al. [13] found that OSA reduced the AERP and increased AF inducibility at the 3rd month in a chronic OSA model. Nevertheless, the authors only tested at the 3rd month. Our study results indicated that the AERP decreased gradually from the 4^th^ week, and the AF inducibility was increased simultaneously, which confirmed that OSA promoted AF occurrence. Moreover, with the extension of chronic OSA modeling time, the waveform of AF tended to be smaller and fragmented, AF was easy to induce and more difficult to terminate. In addition, we observed a certain number of spontaneous AF events during the electrophysiological test every four weeks. However, due to a short period of observation, only a few automatic AF episodes were recorded. Notably, trends in the values for worsening arterial blood gases were consistent with the electrophysiological changes. Although several previous studies measured arterial blood gases before and after apnea [6, 13], in the present study, we observed that PO_2_ and pH gradually decreased and PCO_2_ increased beginning 4^th^ week after modeling started. Hypoxemia and hypercapnia progressively worsened over time, which was similar to the results of obstructive sleep apnea syndrome patients [26]. An unexpected finding was the extent to which significant hypoxemia, hypercapnia, and lower pH values were observed even if there was no airway obstruction in the OSA group.

### OSA increased LF and LF/HF

Cardiac autonomic nervous system (CANS) activity could be measured indirectly by HRV [27]. Frequency-domain indices of HRV included VLF, LF, HF, and the LF/HF ratio. Of these, LF reflects sympathetic activity, HF reflects parasympathetic influences activity, and the LF/HF ratio reflects the sympathetic/parasympathetic balance [16]. Several clinical studies have investigated CANS changes by evaluating the HRV, and the results showed that higher LF and LF/HF ratios were observed in moderate and severe OSA patients [28, 29]. Although we found that the LFnu and LFnu/HFnu were increased from the 4^th^ week to the 12^th^ week, no marked variation was observed in HFnu. Of note, to avoid potential effects of anesthesia, all HRV indicators were repeatedly collected in the nonanesthetized condition. These conclusions are in accord with previous clinical study studies indicating that LF and the LF/HF ratio were both upregulated in OSA patients [28]. Previous clinical and basic studies had confirmed that sympathetic hyperactivity was closely related to AF inducibility [30–32]. Similarly, in our study, we found that HRV variation was consistent with atrial electrophysiological changes.

### Enhanced atrial neural remodeling in the chronic model of OSA

Some clinical and experimental studies have demonstrated that atrial sympathetic nerve sprouting or cholinergic innervation could increase AF susceptibility [33, 34]. In the current study, the TH-, CHAT-, and PGP9.5-positive nerve densities and protein expressions are remarkably higher in the left atrium than in the control group. These results might explain why both sympathetic and parasympathetic neurons were involved in atrial neural remodeling in OSA. Although previous studies have confirmed that TH- and CHAT-positive nerve fiber densities are increased in a chronic model of OSA, no literature is available on the expressions of atrial TH, CHAT, and PGP9.5 in OSA patients [11, 35]. It has been reported that NGF is an important neurotrophic factor that supported the differentiation, maturation, and survival of sympathetic neurons [36]. C-Fos is a rapid indicator of the comprehensive activation of neural elements [37]. Several studies have confirmed that NGF and c-Fos promote autonomic nerve sprouting in the atrium [33, 38–40]. For verification, we found that the protein expressions of NGF and c-Fos were both increased in the left atrium. This means that evident neural remodeling did occur in the left atrium of the OSA group. A previous study confirmed that Cx43 is one of the primary connexins that is characteristic of transmission velocity and anisotropic conductive properties in the atrium [41]. Moreover, Cx43 participates in tissue inflammation and repair, and lower expression of connexins was related to enhanced AF susceptibility [24, 42]. However, the association between Cx43 and the OSA-induced AF has not been well explored.

Moreover, evidence from other experimental animal studies showed that Cx43 reduced susceptibility to sympathetic AF [23, 43]. In the present study, the mRNA expression levels of Cx43 in the OSA group decreased, and immunohistochemical results showed a significant decrease in Cx43 but not in Cx40. We hypothesized that reduced Cx43 expression was correlated with OSA-induced AF. When these two connexin proteins were combined, we found that the Cx43/Cx40 ratio decreased in the OSA group. Furthermore, the sparse distribution of Cx43 provides a solid foundation for OSA-induced AF. However, this differs from the findings presented by Zhao et al. [13], who showed no apparent changes in Cx40 and Cx43 in the total bilateral atria, appendage, and free wall tissues.

### Enhanced LSG activity facilitated AF in the chronic model of OSA

To the best of our knowledge, this is the first study to evaluate the role of the LSG and LVN in an OSA-associated AF model. In the present study, we observed spontaneous AF in the OSA group. Previous studies have confirmed that apnea could significantly alter the autonomic nervous system [44]. Furthermore, repeated hypoxia and CO_2_ retention stimulate central and peripheral chemoreceptors, increasing sympathetic nervous system activities [35].

Nevertheless, there is no related description regarding whether the stellate ganglia or cervical vagus nerve participates in the OSA-induced AF. Moreover, these neural activity findings were further confirmed via immunohistochemical staining of nerve fiber density in the LSG and LVN. Our study found that the expressions of TH and PGP9.5 were remarkably increased in the LSG compared to the control group, but no neural distribution changes were found in the LVN. Simultaneously, we first reported that the increased protein expressions of NGF and c-Fos in the LSG, which confirmed neural innervation in the LSG and further demonstrated that sympathetic nerve remodeling played a critical role in OSA. Moreover, we found that AF inducibility was markedly increased under LSG stimulation without changing the stimulus threshold. The IKACh channel is a heterotetramer of Kir3.1 and Kir3.4, the two inwardly rectifying potassium channel proteins encoded by KCNJ3 and KCNJ5, respectively [45]. P Kovoor et al. [46] found that AF was no longer induced by vagus nerve stimulation in a mouse model in which the I(KACh) gene was knocked out. In the present study, we found that the mRNA expression of Kir3.1 was significantly decreased in the chronic OSA group. We believe that in some cases, the mRNA expression level of Kir3.1 influences the IKACh channel, but more experiments are needed to explore the possible mechanism.

### Clinical implication

Current therapeutic strategies have less than the ideal therapeutic effect for the treatment of OSA-related AF. Evidence from an experimental study showed that metoprolol prevented OSA-induced AF by inhibiting structural and sympathetic nerve remodeling of the atrium [47]. However, various autonomic nerve regulation approaches were used to treat AF, such as renal denervation, ganglionated plexi ablation, low-level vagal nerve stimulation, and carotid body ablation [48]. Here we provided an evidence that the LSG significantly facilitated AF in the chronic model of OSA, which provided a preliminary basis for autonomic nervous intervention in OSA treatment with AF.

### Limitations

The present study has several limitations. First, we did not investigate the effect of LSG-blocked atrial nerve distribution. Based on previous research, LSG stimulation induced sympathetic and parasympathetic hyperinnervation in the atrium [49]. Therefore, we consider that changes in the LSG will affect atrial neural distribution and remodeling. Additionally, high-frequency stimulation of the LSG and LVN was conducted to detect the AF inducibility. We found that OSA-induced AF was closely related to the hyperactivity of LSG. Second, we failed to record LSG and LVN neural activity during chronic OSA. However, we used the HRV to show the CANS changes indirectly. Third, previous research has confirmed that the bilateral stellate ganglion and vagus nerve have different atrial remodeling roles. To ensure accurate and stable recorded neural activity, we only focused on the left stellate ganglia and cervical vagus nerve, which may not fully reflect the extrinsic autonomic nervous system. We considered that the mRNA expression level of Kir3.1 was not always consistent with the current IKACh. Meanwhile, we didn’t perform the path clamp or other experimental evidences to explain these results. The possible mechanisms need further investigation. Finally, a small sample size may have impacted the conclusions. Nevertheless, our study provided some useful implications for better understanding the neural remodeling mechanism of a chronic model of OSA induced-AF.

## Conclusions

In summary, our study found that OSA significantly enhanced neural activity and remodeling of LSG, and hyperactivity of LSG might accelerate left atrial neural remodeling to increase AF inducibility.

## Supporting information

S1 File (DOCX)

S1 Raw images (PDF)
