# Supplementary material for: Enhanced atrial internal-external neural remodeling facilitates atrial fibrillation in the chronic obstructive sleep apnea model
Source: PLoS One. 2021 Feb 19;16(2):e0247308. doi: 10.1371/journal.pone.0247308 (PMC7895341; doi:10.1371/journal.pone.0247308)
Supplement: S1 File — (DOCX) [file pone.0247308.s001.docx]

|  | | |  |  |
| --- | --- | --- | --- | --- |
| **Table 1. Arterial blood gases analysis** | | |  |  |
| *preapnea vs.postapnea* | | | *Mean±SD* | *P Value* |
| *PO2(mmHg)* | | | *95.62 ± 2.51 vs. 41.66 ± 2.15* | *0.0001* |
| *PCO2(mmHg)* | | | *42.05 ± 1.72 vs. 50.99 ± 2.38* | *0.0062* |
| *pH* | | | *7.38 ± 0.04 vs. 7.29 ± 0.02* | *0.0319* |
|  | | |  |  |
| **Table 2. Arterial blood gases analysis of PO_2_** | | |  |  |
| *Control vs. OSA（PO_2_，mmHg)* | | | *Mean±SD* | *P Value* |
| *BS* | | | *98.11±0.66 vs. 97.81±0.37* | *0.5429* |
| *4W* | | | *96.62±0.79 vs. 90.72±0.49* | *0.0028* |
| *8W* | | | *98.06±0.59 vs. 86.01±0.67* | *<0.0001* |
| *12W* | | | *97.75±0.56 vs. 78.91±2.05* | *0.0046* |
| **Table 3. Arterial blood gases analysis of PCO_2_** | | |  |  |
| *Control vs. OSA（PCO_2_，mmHg)* | | | *Mean±SD* | *P Value* |
| *BS* | | | *39.76±0.69 vs. 39.88±1.26* | *0.8884* |
| *4W* | | | *40.65±0.73 vs. 43.09±0.52* | *0.0237* |
| *8W* | | | *40.85±0.40vs. 46.62±1.13* | *0.0209* |
| *12W* | | | *41.53±1.01 vs. 53.42±1.91* | *0.0094* |
|  |  |  |  |  |
| **Table 4. Arterial blood gases analysis of pH** | | |  |  |
| *Control vs. OSA（pH)* | | | *Mean±SD* | *P Value* |
| *BS* | | | *7.36±0.32 vs. 7.35±0.02* | *0.5808* |
| *4W* | | | *7.37±0.02 vs. 7.27±0.01* | *0.0142* |
| *8W* | | | *7.37±0.04 vs. 7.23±0.02* | *0.0401* |
| *12W* | | | *7.36±0.02 vs. 7.19±0.01* | *0.0009* |
|  | | |  |  |
| **Table 5. Atrial electrophysiology analysis** | | |  |  |
| *Control vs. OSA（AERP，ms)* | | | *Mean±SD* | *P Value* |
| *BS* | | | *138.33 ± 20.21 vs. 140.56±16.71* | *>0.9999* |
| *4W* | | | *131.81 ± 5.45 vs. 109.16 ±23.47* | *0.0051* |
| *8W* | | | *130.56±16.48 vs. 98.00±20.21* | *0.001* |
| *12W* | | | *131.43± 8.018 vs. 80.24 ± 11.34* | *<0.0001* |
|  | | |  |  |
| **Table 6. Atrial electrophysiology analysis** | | |  |  |
| *Control+APNEA vs. OSA+APNEA（AERP，ms)* | | | *Mean±SD* | *P Value* |
| *BS* | | | *119.0 ± 4.18 vs. 115.53 ± 17.56* | *0.9699* |
| *4W* | | | *120.00 ± 6.45 vs. 86.67 ± 9.01* | *<0.0001* |
| *8W* | | | *116.43 ± 14.35 vs. 82.75 ± 8.96* | *0.0027* |
| *12W* | | | *114.23 ± 8.38 vs. 66.30 ± 9.68* | *<0.0001* |
|  | | |  |  |
| **Table 7. Atrial electrophysiology analysis** | | |  |  |
| *Control（AERP，ms)* | | | *Mean±SD* | *P Value* |
| *BS vs. 4W* | | | *138.33 ± 20.21 vs. 131.82 ± 5.46* | *0.8969* |
| *BS vs. 8W* | | | *138.33 ± 20.21 vs. 130.56±16.48* | *0.9955* |
| *BS vs. 12W* | | | *138.33 ± 20.21 vs. 131.43± 8.018* | *0.9988* |
| *4W vs. 8W* | | | *131.82 ± 5.46 vs. 130.56±16.48* | *>0.9999* |
| *4W vs. 12W* | | | *131.82 ± 5.46 vs. 131.43± 8.018* | *>0.9999* |
| *8W vs. 12W* | | | *130.56±16.48 vs. 131.43± 8.018* | *>0.9999* |
|  | | |  |  |
| **Table 8. Atrial electrophysiology analysis** | | |  |  |
| *OSA（AERP，ms)* | | | *Mean±SD* | *P Value* |
| *BS vs. 4W* | | | *140.56±16.71 vs. 109.17±23.47* | 0.0025 |
| *BS vs. 8W* | | | *140.56±16.71 vs. 98.00±20.21* | 0.0002 |
| *BS vs. 12W* | | | *140.56±16.71 vs. 80.24 ± 11.34* | <0.0001 |
| *4W vs. 8W* | | | *109.17±23.47 vs. 98.00±20.21* | 0.7328 |
| *4W vs. 12W* | | | *109.17±23.47 vs. 80.24 ± 11.34* | 0.0002 |
| *8W vs. 12W* | | | *98.00±20.21 vs. 80.24 ± 11.34* | 0.0367 |
|  | | |  |  |
| **Table 9. Atrial electrophysiology analysis** | | |  |  |
| *Control+APNEA（AERP，ms)* | | | *Mean±SD* | *P Value* |
| *BS vs. 4W* | | | *119.0 ± 4.18 vs. 120.00 ± 6.45* | *0.9997* |
| *BS vs. 8W* | | | *119.0 ± 4.18 vs. 116.43 ± 14.35* | *0.9996* |
| *BS vs. 12W* | | | *119.0 ± 4.18 vs. 114.23 ± 8.38* | *0.9096* |
| *4W vs. 8W* | | | *120.00 ± 6.45 vs. 116.43 ± 14.35* | *0.9947* |
| *4W vs. 12W* | | | *120.00 ± 6.45 vs. 114.23 ± 8.38* | *0.7132* |
| *8W vs. 12W* | | | *116.43 ± 14.35 vs. 114.23 ± 8.38* | *>0.9999* |
|  | | |  |  |
| **Table 10. Atrial electrophysiology analysis** | | |  |  |
| *OSA+APNEA（AERP，ms)* | | | *Mean±SD* | *P Value* |
| *BS vs. 4W* | | | *115.53 ± 17.56vs. 86.67 ± 9.01* | *0.0029* |
| *BS vs. 8W* | | | *115.53 ± 17.56 vs. 82.75 ± 8.96* | *<0.0001* |
| *BS vs. 12W* | | | *115.53 ± 17.56 vs. 66.30 ± 9.68* | *<0.0001* |
| *4W vs. 8W* | | | *86.67 ± 9.01 vs. 82.75 ± 8.96* | *0.8324* |
| *4W vs. 12W* | | | *86.67 ± 9.01 vs. 66.30 ± 9.68* | *0.0005* |
| *8W vs. 12W* | | | *82.75 ± 8.96 vs. 66.30 ± 9.68* | *0.0005* |
|  | | |  |  |
| **Table 11. WOV analysis** | | |  |  |
| *Control+APNEA vs. OSA+APNEA（WOV，ms)* | | | *Mean±SD* | *P Value* |
| *BS* | | | *11.25 ± 4.78 vs. 8.75 ± 2.5* | *0.9906* |
| *4W* | | | *6.67 ± 2.89 vs. 24 ± 8.94* | *0.0011* |
| *8W* | | | *11.00 ± 4.18 vs. 29 ± 4.18* | *<0.0001* |
| *12W* | | | *10.00 ± 7.07 vs. 37 ± 5.70* | *<0.0001* |
|  | | |  |  |
| **Table 12. WOV analysis** | | |  |  |
| *Control+APNEA（WOV，ms)* | | | *Mean±SD* | *P Value* |
| *BS vs. 4W* | | | *11.25 ± 4.78 vs. 6.67 ± 2.89* | *0.8837* |
| *BS vs. 8W* | | | *11.25 ± 4.78 vs. 11.00 ± 4.18* | *>0.9999* |
| *BS vs. 12W* | | | *11.25 ± 4.78vs. 10.00 ± 7.07* | *0.9998* |
| *4W vs. 8W* | | | *6.67 ± 2.89 vs. 11.00 ± 4.18* | *0.889* |
| *4W vs. 12W* | | | *6.67 ± 2.89 vs. 10.00 ± 7.07* | *0.9725* |
| *8W vs. 12W* | | | *11.00 ± 4.18 vs. 10.00 ± 7.07* | *>0.9999* |
|  | | |  |  |
| **Table 13. WOV analysis** | | |  |  |
| *OSA+APNEA（WOV，ms)* | | | *Mean±SD* | *P Value* |
| *BS vs. 4W* | | | *8.75 ± 2.5 vs. 24 ± 8.94* | *0.0019* |
| *BS vs. 8W* | | | *8.75 ± 2.5 vs. 29 ± 4.18* | *<0.0001* |
| *BS vs. 12W* | | | *8.75 ± 2.5 vs. 37 ± 5.70* | *<0.0001* |
| *4W vs. 8W* | | | *24 ± 8.94 vs. 29 ± 4.18* | *0.6861* |
| *4W vs. 12W* | | | *24 ± 8.94 vs. 37 ± 5.70* | *0.0057* |
| *8W vs. 12W* | | | *29 ± 4.18 vs. 37 ± 5.70* | *0.1845* |

|  |  |  |  |
| --- | --- | --- | --- |
| **Table 14. AF inducibility analysis** |  |  |  |
| Data analyzed | AF | NON-AF | Total |
| Control-BS | 2 | 48 | 50 |
| OSA-BS | 1 | 49 | 50 |
| Total | 3 | 97 | 100 |
|  |  |  |  |
| P value and statistical significance |  |  |  |
| Test | Fisher's exact test |  |  |
| P value | >0.9999 |  |  |
| P value summary | ns |  |  |
| One- or two-sided | Two-sided |  |  |
| Statistically significant (P < 0.05)? | No |  |  |
|  |  |  |  |
| **Table 15. AF inducibility analysis** |  |  |  |
| Data analyzed | AF | NON-AF | Total |
| Control-4W | 3 | 47 | 50 |
| OSA-4W | 11 | 39 | 50 |
| Total | 14 | 86 | 100 |
|  |  |  |  |
| P value and statistical significance |  |  |  |
| Test | Fisher's exact test |  |  |
| P value | 0.0407 |  |  |
| One- or two-sided | Two-sided |  |  |
| Statistically significant (P < 0.05)? | Yes |  |  |
|  |  |  |  |
| **Table 16. AF inducibility analysis** |  |  |  |
| Data analyzed | AF | NON-AF | Total |
| Control-8W | 3 | 47 | 50 |
| OSA-8W | 21 | 29 | 50 |
| Total | 24 | 76 | 100 |
|  |  |  |  |
| P value and statistical significance |  |  |  |
| Test | Fisher's exact test |  |  |
| P value | <0.0001 |  |  |
| One- or two-sided | Two-sided |  |  |
| Statistically significant (P < 0.05)? | Yes |  |  |
|  |  |  |  |
| **Table 17. AF inducibility analysis** |  |  |  |
| Data analyzed | AF | NON-AF | Total |
| Control-12W | 1 | 49 | 50 |
| OSA-12W | 37 | 13 | 50 |
| Total | 38 | 62 | 100 |
|  |  |  |  |
| P value and statistical significance |  |  |  |
| Test | Fisher's exact test |  |  |
| P value | <0.0001 |  |  |
| One- or two-sided | Two-sided |  |  |
| Statistically significant (P < 0.05)? | Yes |  |  |
|  |  |  |  |
| **Table 18. AF inducibility analysis** |  |  |  |
| Data analyzed | AF | NON-AF | Total |
| Control+APNEA-BS | 32 | 18 | 50 |
| OSA+APNEA-BS | 27 | 23 | 50 |
| Total | 59 | 41 | 100 |
|  |  |  |  |
| P value and statistical significance |  |  |  |
| Test | Fisher's exact test |  |  |
| P value | 0.4162 |  |  |
| P value summary | ns |  |  |
| One- or two-sided | Two-sided |  |  |
| Statistically significant (P < 0.05)? | No |  |  |
|  |  |  |  |
| **Table 19. AF inducibility analysis** |  |  |  |
| Data analyzed | AF | NON-AF | Total |
| Control+APNEA-4W | 35 | 15 | 50 |
| OSA+APNEA-4W | 50 | 0 | 50 |
| Total | 85 | 15 | 100 |
|  |  |  |  |
| P value and statistical significance |  |  |  |
| Test | Fisher's exact test |  |  |
| P value | <0.0001 |  |  |
| One- or two-sided | Two-sided |  |  |
| Statistically significant (P < 0.05)? | Yes |  |  |
|  |  |  |  |
| **Table 20. AF inducibility analysis** |  |  |  |
| Data analyzed | AF | NON-AF | Total |
| Control+APNEA-8W | 26 | 24 | 50 |
| OSA+APNEA-8W | 50 | 0 | 50 |
| Total | 76 | 24 | 100 |
|  |  |  |  |
| P value and statistical significance |  |  |  |
| Test | Fisher's exact test |  |  |
| P value | <0.0001 |  |  |
| One- or two-sided | Two-sided |  |  |
| Statistically significant (P < 0.05)? | Yes |  |  |
|  |  |  |  |
| **Table 21. AF inducibility analysis** |  |  |  |
| Data analyzed | AF | NON-AF | Total |
| Control+APNEA-12W | 31 | 19 | 50 |
| OSA+APNEA-12W | 50 | 0 | 50 |
| Total | 81 | 19 | 100 |
|  |  |  |  |
| P value and statistical significance |  |  |  |
| Test | Fisher's exact test |  |  |
| P value | <0.0001 |  |  |
| One- or two-sided | Two-sided |  |  |
|  |  |  |  |
|  |  |  |  |

| **Table 22. HRV analysis** |  |  |
| --- | --- | --- |
| *Control vs. OSA（LFnu)* | *Mean±SD* | *P Value* |
| *BS* | *34.55 ± 11.19 vs. 28.50 ± 7.22* | *0.7372* |
| *4W* | *33.48 ± 5.73 vs. 52.00 ± 14.16* | *0.0148* |
| *8W* | *35.12 ± 7.15 vs. 56.23 ± 8.07* | *0.0054* |
| *12W* | *32.25 ± 5.54 vs. 51.28 ± 6.24* | *0.0121* |
|  |  |  |
| **Table 23. HRV analysis** |  |  |
| *Control（LFnu)* | *Mean±SD* | *P Value* |
| *BS vs. 4W* | *34.55 ± 11.19 vs. 33.47 ± 5.73* | *>0.9999* |
| *BS vs. 8W* | *34.55 ± 11.19 vs. 35.12 ± 7.15* | *>0.9999* |
| *BS vs. 12W* | *34.55 ± 11.19 vs. 32.25 ± 5.54* | *0.9989* |
| *4W vs. 8W* | *33.47 ± 5.73 vs. 35.12 ± 7.15* | *0.9998* |
| *4W vs. 12W* | *33.47 ± 5.73 vs. 32.25 ± 5.54* | *>0.9999* |
| *8W vs. 12W* | *35.12 ± 7.15 vs. 32.25 ± 5.54* | *0.9963* |
|  |  |  |
| **Table 24. HRV analysis** |  |  |
| *OSA（LFnu)* | *Mean±SD* | *P Value* |
| *BS vs. 4W* | *28.50 ± 7.22 vs. 51.99 ± 14.16* | *0.0016* |
| *BS vs. 8W* | *28.50 ± 7.22 vs. 56.25 ± 8.07* | *0.0002* |
| *BS vs. 12W* | *28.50 ± 7.22 vs. 51.28 ± 6.24* | *0.0022* |
| *4W vs. 8W* | *51.99 ± 14.16 vs. 56.25 ± 8.07* | *0.9715* |
| *4W vs. 12W* | *51.99 ± 14.16 vs. 51.28 ± 6.24* | *>0.9999* |
| *8W vs. 12W* | *56.25 ± 8.07 vs. 51.28 ± 6.24* | *0.9409* |
|  |  |  |
| **Table 25. HRV analysis** |  |  |
| *Control vs. OSA（HFnu)* | *Mean±SD* | *P Value* |
| *BS* | *60.97 ± 9.77 vs. 61.26 ± 11.35* | *>0.9999* |
| *4W* | *60.24 ± 3.79 vs. 44.91 ± 11.64* | *0.0581* |
| *8W* | *56.42 ± 6.29 vs. 46.28 ± 11.69* | *0.3378* |
| *12W* | *58.87 ± 4.55 vs. 52.88 ± 11.71* | *0.7878* |
|  |  |  |
| **Table 26. HRV analysis** |  |  |
| *Control（HFnu)* | *Mean±SD* | *P Value* |
| *BS vs. 4W* | *60.97 ± 9.77 vs. 60.24 ± 3.79* | *>0.9999* |
| *BS vs. 8W* | *60.97 ± 9.77 vs. 56.42 ± 6.29* | *0.9127* |
| *BS vs. 12W* | *60.97 ± 9.77 vs. 58.87 ± 4.55* | *0.9994* |
| *4W vs. 8W* | *60.24 ± 3.79 vs. 56.42 ± 6.29* | *0.2721* |
| *4W vs. 12W* | *60.24 ± 3.79 vs. 58.87 ± 4.55* | *0.9966* |
| *8W vs. 12W* | *56.42 ± 6.29 vs. 58.87 ± 4.55* | *0.9522* |
|  |  |  |
| **Table 27. HRV analysis** |  |  |
| *OSA（HFnu)* | *Mean±SD* | *P Value* |
| *BS vs. 4W* | *61.26 ± 11.35 vs. 44.91 ± 11.64* | *0.6550* |
| *BS vs. 8W* | *61.26 ± 11.35 vs. 46.28 ± 11.69* | *0.7071* |
| *BS vs. 12W* | *61.26 ± 11.35 vs. 52.88 ± 11.71* | *0.8882* |
| *4W vs. 8W* | *44.91 ± 11.64 vs. 46.28 ± 11.69* | *>0.9999* |
| *4W vs. 12W* | *44.91 ± 11.64 vs. 52.88 ± 11.71* | *0.8387* |
| *8W vs. 12W* | *46.28 ± 11.69 vs. 52.88 ± 11.71* | *0.9884* |
|  |  |  |
| **Table 28. HRV analysis** |  |  |
| *Control vs. OSA（LFnu/HnuF)* | *Mean±SD* | *P Value* |
| *BS* | *0.61 ± 0.35 vs. 0.50 ± 0.22* | *0.6184* |
| *4W* | *0.56 ± 0.12 vs. 1.29 ± 0.64* | *0.0141* |
| *8W* | *0.64 ± 0.19 vs. 1.31 ± 0.55* | *0.0187* |
| *12W* | *0.55 ± 0.10 vs. 1.14 ± 0.35* | *0.0300* |
|  |  |  |
| **Table 29. HRV analysis** |  |  |
| *Control（LFnu/HFnu)* | *Mean±SD* | *P Value* |
| *BS vs. 4W* | *0.61 ± 0.35 vs. 0.56 ± 0.12* | *>0.9999* |
| *BS vs. 8W* | *0.61 ± 0.35 vs. 0.64 ± 0.19* | *>0.9999* |
| *BS vs. 12W* | *0.61 ± 0.35 vs. 0.55 ± 0.10* | *>0.9999* |
| *4W vs. 8W* | *0.56 ± 0.12 vs. 0.64 ± 0.19* | *0.9997* |
| *4W vs. 12W* | *0.56 ± 0.12 vs. 0.55 ± 0.10* | *>0.9999* |
| *8W vs. 12W* | *0.64 ± 0.19 vs. 0.55 ± 0.10* | *0.9995* |
|  |  |  |
| **Table 30. HRV analysis** |  |  |
| *OSA（LFnu/HFnu)* | *Mean±SD* | *P Value* |
| *BS vs. 4W* | *0.50 ± 0.22 vs. 1.29 ± 0.64* | *0.0152* |
| *BS vs. 8W* | *0.50 ± 0.22 vs. 1.31 ± 0.55* | *0.0139* |
| *BS vs. 12W* | *0.50 ± 0.22 vs. 1.14 ± 0.35* | *0.0498* |
| *4W vs. 8W* | *1.29 ± 0.64 vs. 1.31 ± 0.55* | *>0.9999* |
| *4W vs. 12W* | *1.29 ± 0.64 vs. 1.14 ± 0.35* | *0.9922* |
| *8W vs. 12W* | *1.31 ± 0.55 vs. 1.14 ± 0.35* | *0.9810* |
|  |  |  |
| **Table 31. LA diameter analysis** |  |  |
| *Control vs. OSA（LA diameter,mm)* | *Mean±SD* | *P Value* |
| *BS* | *25.75 ± 1.54 vs. 23.12 ± 1.30* | *0.0777* |
| *4W* | *26.33 ± 1.44 vs. 25.07 ± 1.25* | *0.5422* |
| *8W* | *24.55 ± 1.63 vs. 28.91 ± 2.01* | *0.0234* |
| *12W* | *26.11 ± 1.11 vs. 30.64 ± 1.76* | *0.008* |
|  |  |  |
| **Table 32. LA diameter analysis** |  |  |
| *Control（LA diameter,mm)* | *Mean±SD* | *P Value* |
| *BS vs. 4W* | *25.75 ± 1.54 vs. 26.33 ± 1.44* | *0.9202* |
| *BS vs. 8W* | *25.75 ± 1.54 vs. 24.55 ± 1.63* | *0.4071* |
| *BS vs. 12W* | *25.75 ± 1.54 vs. 26.11 ± 1.11* | *0.9336* |
| *4W vs. 8W* | *26.33 ± 1.44 vs. 24.55 ± 1.63* | *0.1427* |
| *4W vs. 12W* | *26.33 ± 1.44 vs. 26.11 ± 1.11* | *0.993* |
| *8W vs. 12W* | *24.55 ± 1.63 vs. 26.11 ± 1.11* | *0.146* |
|  |  |  |
| **Table 33. LA diameter analysis** |  |  |
| *OSA（LA diameter,mm)* | *Mean±SD* | *P Value* |
| *BS vs. 4W* | *23.12 ± 1.30 vs. 25.07 ± 1.25* | *0.2817* |
| *BS vs. 8W* | *23.12 ± 1.30 vs. 28.91 ± 2.01* | *0.0054* |
| *BS vs. 12W* | *23.12 ± 1.30 vs. 30.64 ± 1.76* | *0.0005* |
| *4W vs. 8W* | *25.07 ± 1.25 vs. 28.91 ± 2.01* | *0.0196* |
| *4W vs. 12W* | *25.07 ± 1.25 vs. 30.64 ± 1.76* | *0.0152* |
| *8W vs. 12W* | *28.91 ± 2.01 vs. 30.64 ± 1.76* | *0.138* |
|  |  |  |
| **Table 34. RA diameter analysis** |  |  |
| *Control vs. OSA（RA diameter,mm)* | *Mean±SD* | *P Value* |
| *BS* | *22.36 ± 2.40 vs. 24.77 ± 3.41* | *0.6614* |
| *4W* | *22.95 ± 2.74 vs. 23.98 ± 1.31* | *0.9261* |
| *8W* | *21.82 ± 2.19 vs. 24.32 ± 2.72* | *0.4783* |
| *12W* | *23.32 ± 1.23 vs. 25.42 ± 1.26* | *0.1094* |
|  |  |  |
| **Table 35. RA diameter analysis** |  |  |
| *Control（RA diameter,mm)* | *Mean±SD* | *P Value* |
| *BS vs. 4W* | *22.36 ± 2.40 vs. 22.95 ± 2.74* | *0.9784* |
| *BS vs. 8W* | *22.36 ± 2.40 vs. 21.82 ± 2.19* | *0.9525* |
| *BS vs. 12W* | *22.36 ± 2.40 vs. 23.32 ± 1.23* | *0.8428* |
| *4W vs. 8W* | *22.95 ± 2.74 vs. 21.82 ± 2.19* | *0.9072* |
| *4W vs. 12W* | *22.95 ± 2.74 vs. 23.32 ± 1.23* | *0.9934* |
| *8W vs. 12W* | *21.82 ± 2.19 vs. 23.32 ± 1.23* | *0.1769* |
|  |  |  |
| **Table 36. RA diameter analysis** |  |  |
| *OSA（RA diameter,mm)* | *Mean±SD* | *P Value* |
| *BS vs. 4W* | *24.77 ± 3.41 vs. 23.98 ± 1.31* | *0.9111* |
| *BS vs. 8W* | *24.77 ± 3.41 vs. 24.32 ± 2.72* | *0.9892* |
| *BS vs. 12W* | *24.77 ± 3.41 vs. 25.42 ± 1.26* | *0.9849* |
| *4W vs. 8W* | *23.98 ± 1.31vs. 24.32 ± 2.72* | *0.9757* |
| *4W vs. 12W* | *23.98 ± 1.31 vs. 25.42 ± 1.26* | *0.5111* |
| *8W vs. 12W* | *24.32 ± 2.72 vs. 25.42 ± 1.26* | *0.8259* |
|  |  |  |
| **Table 37. LVEF analysis** |  |  |
| *Control vs. OSA（LVEF,%)* | *Mean±SD* | *P Value* |
| *BS* | *58.80± 2.78 vs. 59.80 ± 1.79* | *0.9471* |
| *4W* | *58.60 ± 1.67 vs. 58.80 ± 1.30* | *0.9993* |
| *8W* | *59.20 ± 2.39 vs. 58.40 ± 2.30* | *0.9755* |
| *12W* | *59.00± 1.41 vs. 57.80 ± 2.17* | *0.8044* |
|  |  |  |
| **Table 38. LVEF analysis** |  |  |
| *Control（LVEF,%)* | *Mean±SD* | *P Value* |
| *BS vs. 4W* | *58.80± 2.78 vs. 58.60 ± 1.67* | *0.9964* |
| *BS vs. 8W* | *58.80± 2.78 vs. 59.20 ± 2.39* | *0.9842* |
| *BS vs. 12W* | *58.80± 2.78 vs. 59.00± 1.41* | *0.9949* |
| *4W vs. 8W* | *58.60 ± 1.67 vs. 59.20 ± 2.39* | *0.9758* |
| *4W vs. 12W* | *58.60 ± 1.67 vs. 59.00± 1.41* | *0.9461* |
| *8W vs. 12W* | *59.20 ± 2.39 vs. 59.00± 1.41* | *0.9964* |
|  |  |  |
| **Table 39. LVEF analysis** |  |  |
| *OSA（LVEF,%)* | *Mean±SD* | *P Value* |
| *BS vs. 4W* | *59.80 ± 1.79 vs. 58.80 ± 1.30* | *0.7586* |
| *BS vs. 8W* | *59.80 ± 1.79 vs. 58.40 ± 2.30* | *0.5088* |
| *BS vs. 12W* | *59.80 ± 1.79 vs. 57.80 ± 2.17* | *0.6126* |
| *4W vs. 8W* | *58.80 ± 1.30vs. 58.40 ± 2.30* | *0.9699* |
| *4W vs. 12W* | *58.80 ± 1.30 vs. 57.80 ± 2.17* | *0.8007* |
| *8W vs. 12W* | *58.40 ± 2.30 vs. 57.80 ± 2.17* | *0.9461* |
|  |  |  |
|  |  |  |
