# Supplementary material for: Enhanced atrial internal-external neural remodeling facilitates atrial fibrillation in the chronic obstructive sleep apnea model
Source: PLoS One. 2021 Feb 19;16(2):e0247308. doi: 10.1371/journal.pone.0247308 (PMC7895341; doi:10.1371/journal.pone.0247308)
Supplement: S1 Raw images — (PDF) [file pone.0247308.s002.pdf]

Raw data——blot

A (Fig 5A, Fig 8E, Fig 8I)

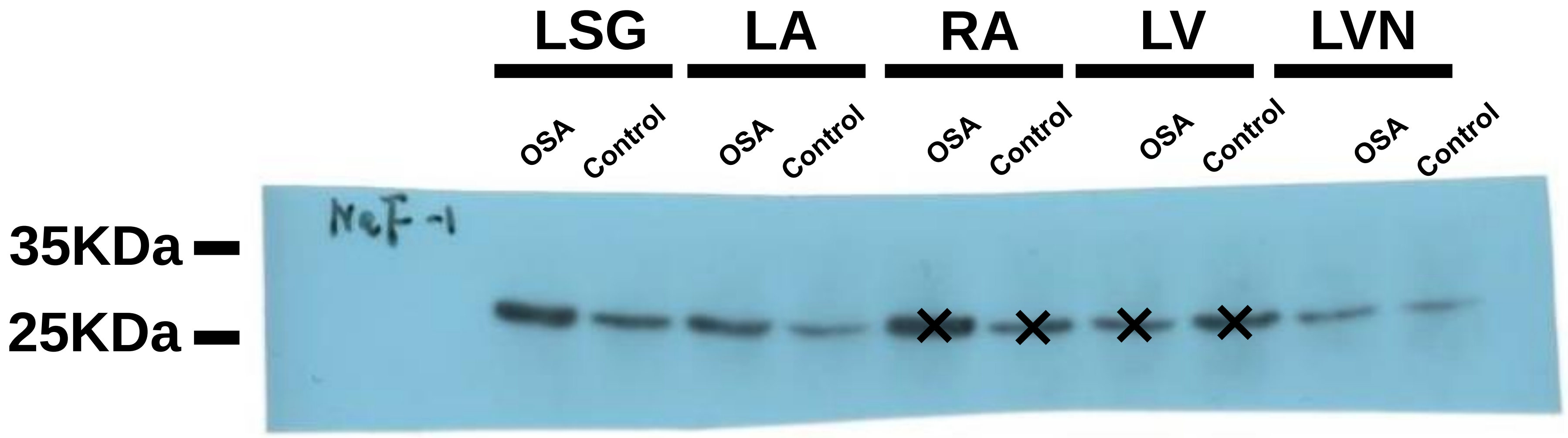

B (Fig 5A, Fig 8E, Fig 8I)

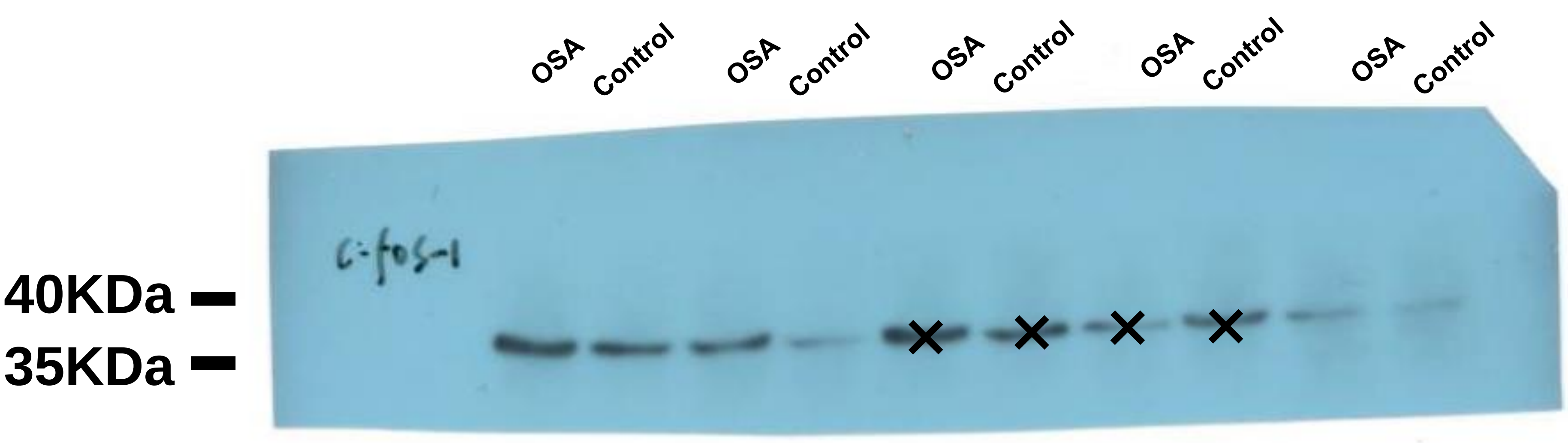

C (Fig 5A, Fig 8E, Fig 8I)

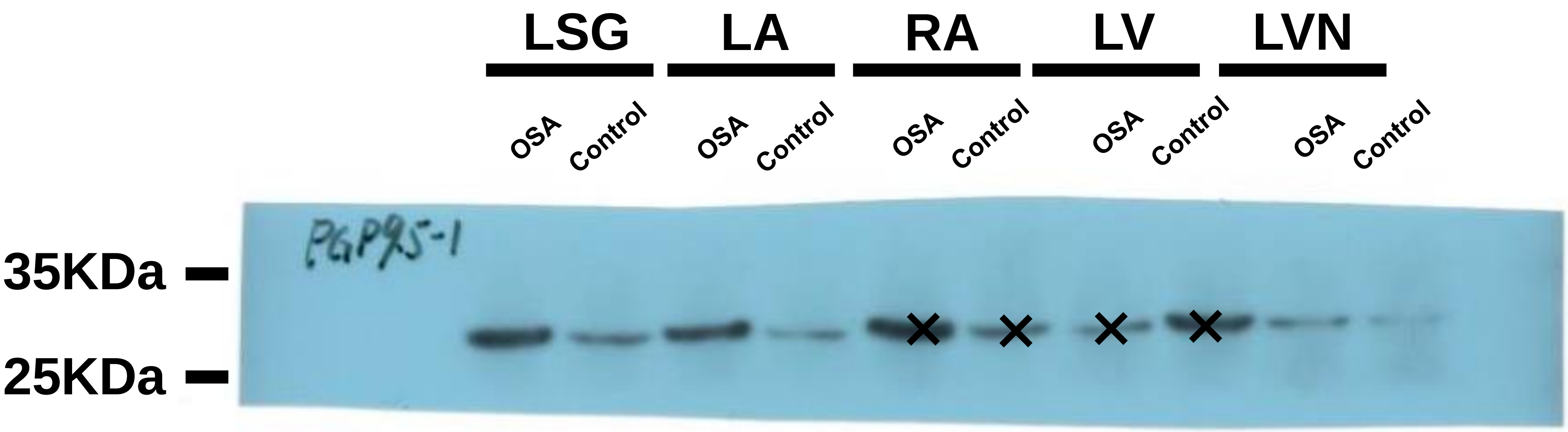

D (Fig 5A, Fig 8E, Fig 8I)

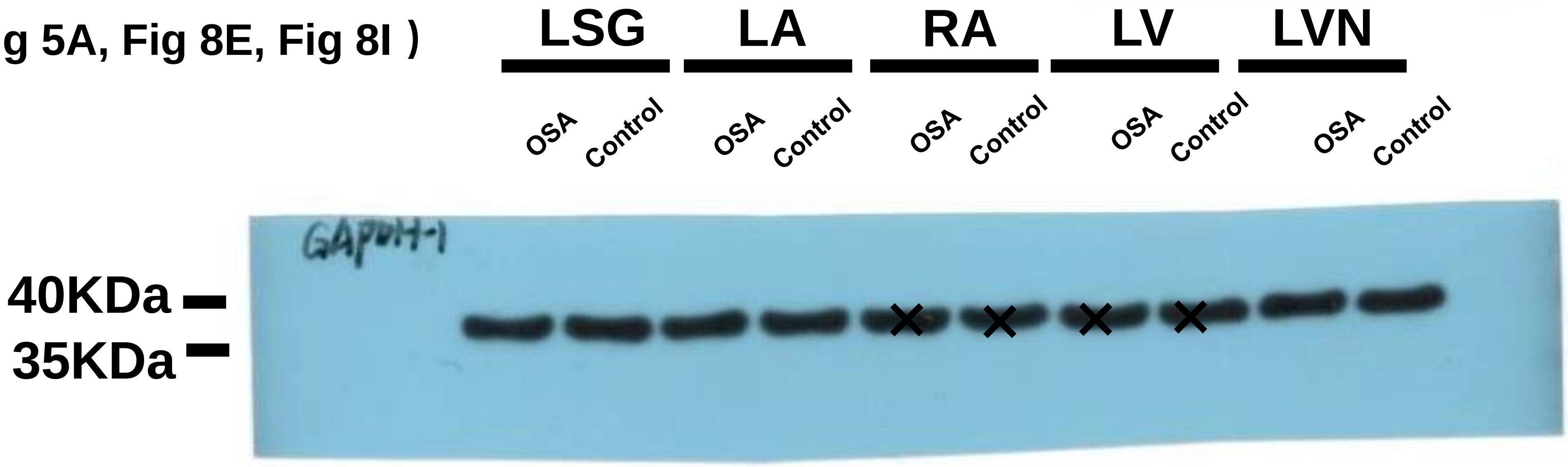

E (Fig 5A)

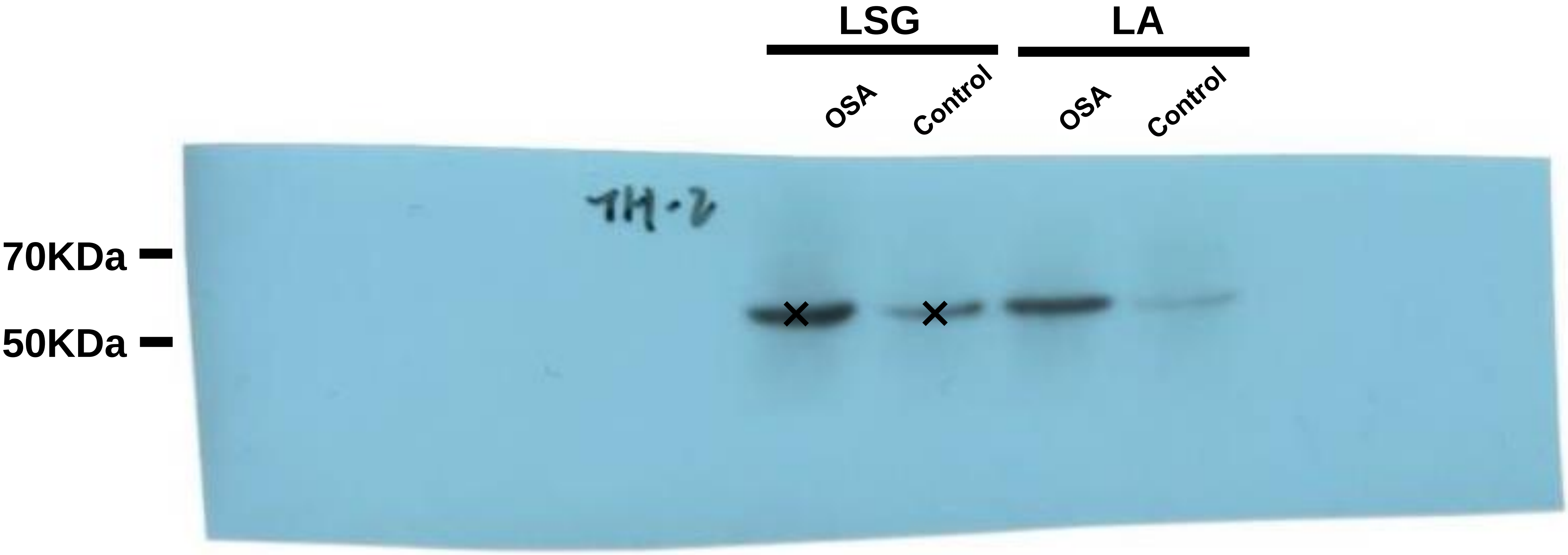

F (Fig 5A)

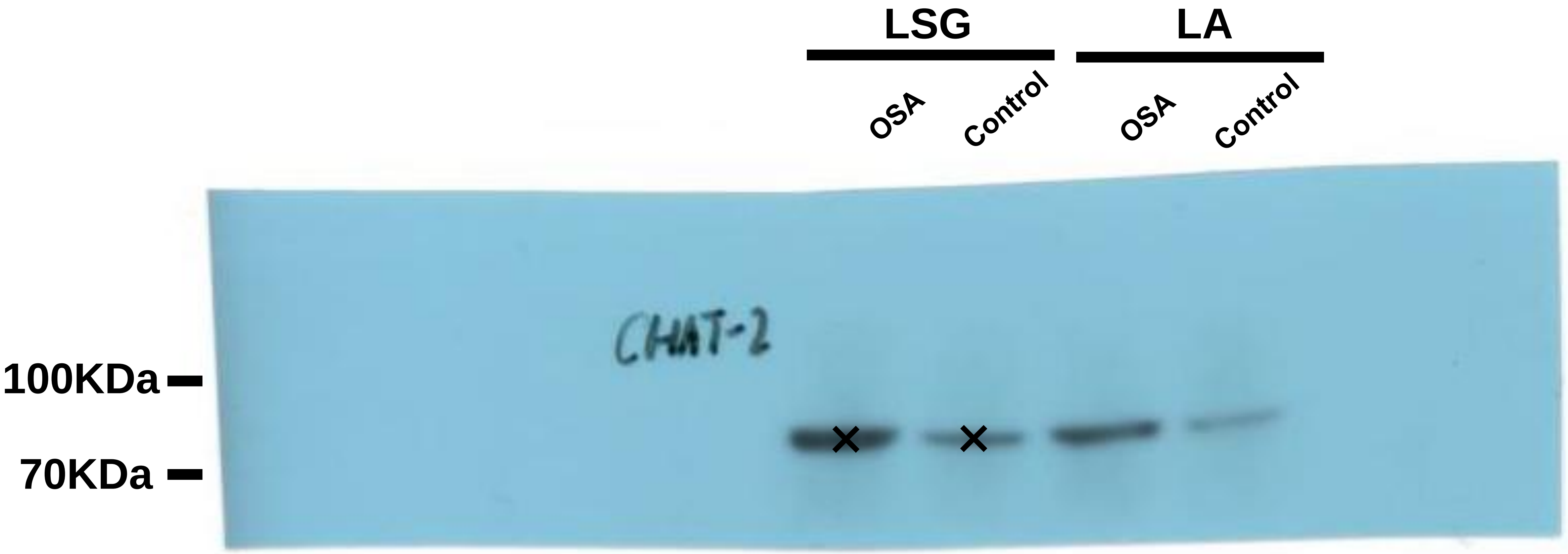

G (Fig 5A)

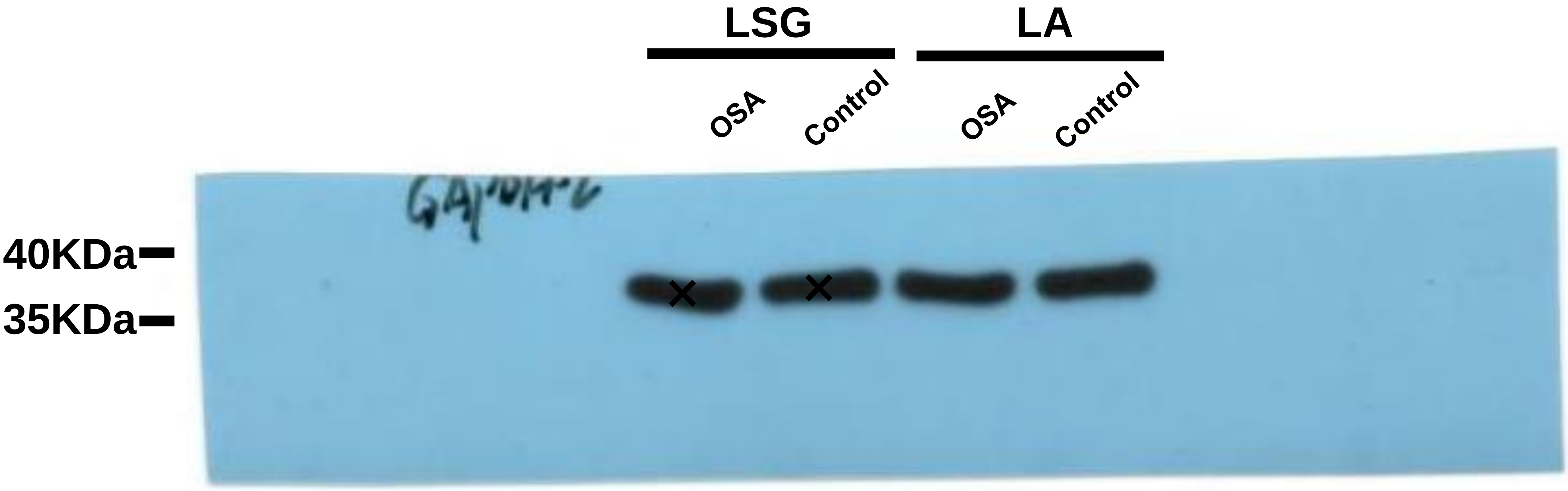

Raw data——Atrial electrophysiology measurements

A (Fig 2D)

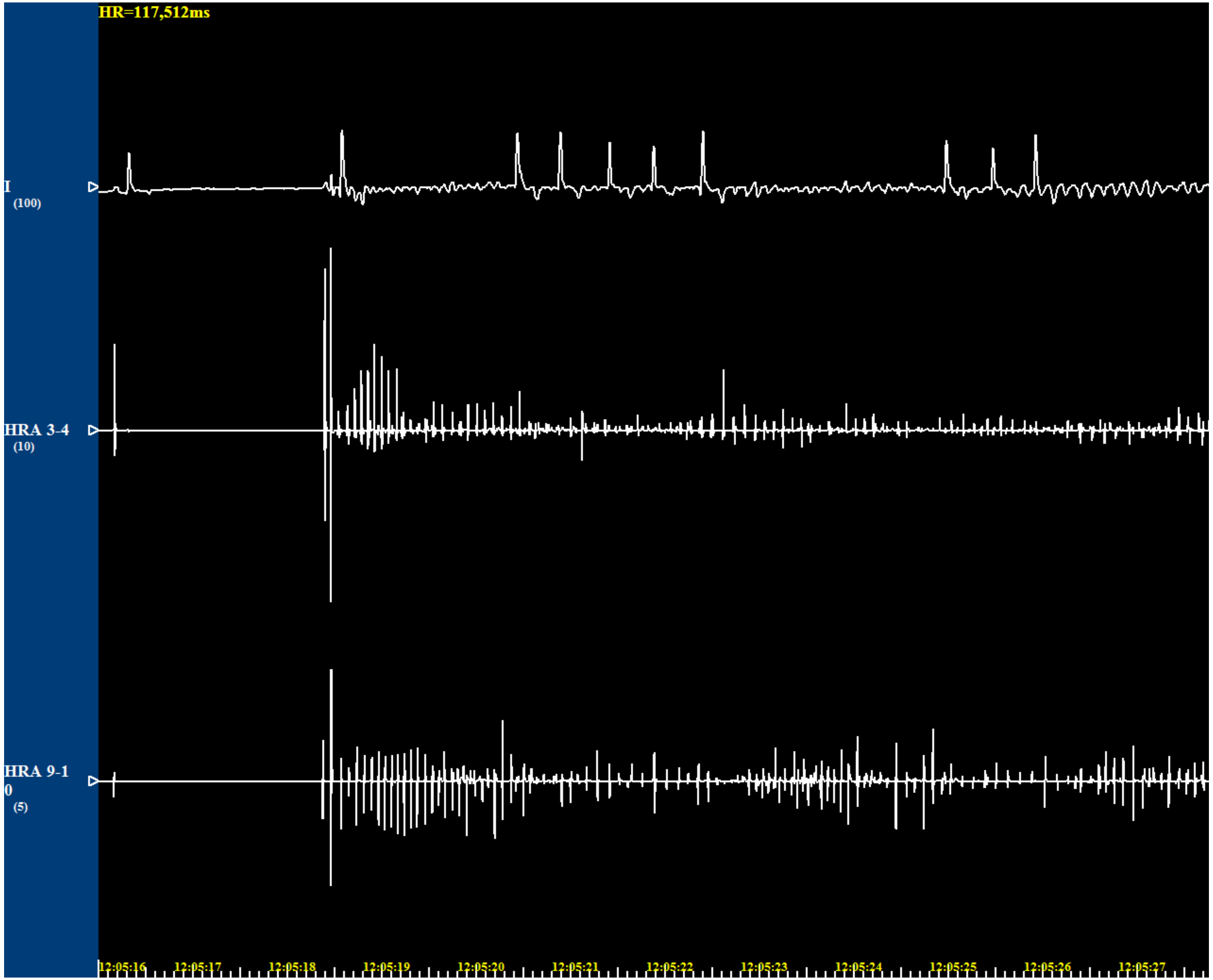

HR=117,512ms
I
(100)
HRA 3-4
(10)
HRA 9-1
0
(5)
12:05:16
12:05:17
12:05:18
12:05:19
12:05:20
12:05:21
12:05:22
12:05:23
12:05:24
12:05:25
12:05:26
12:05:27

B (Fig 2E)

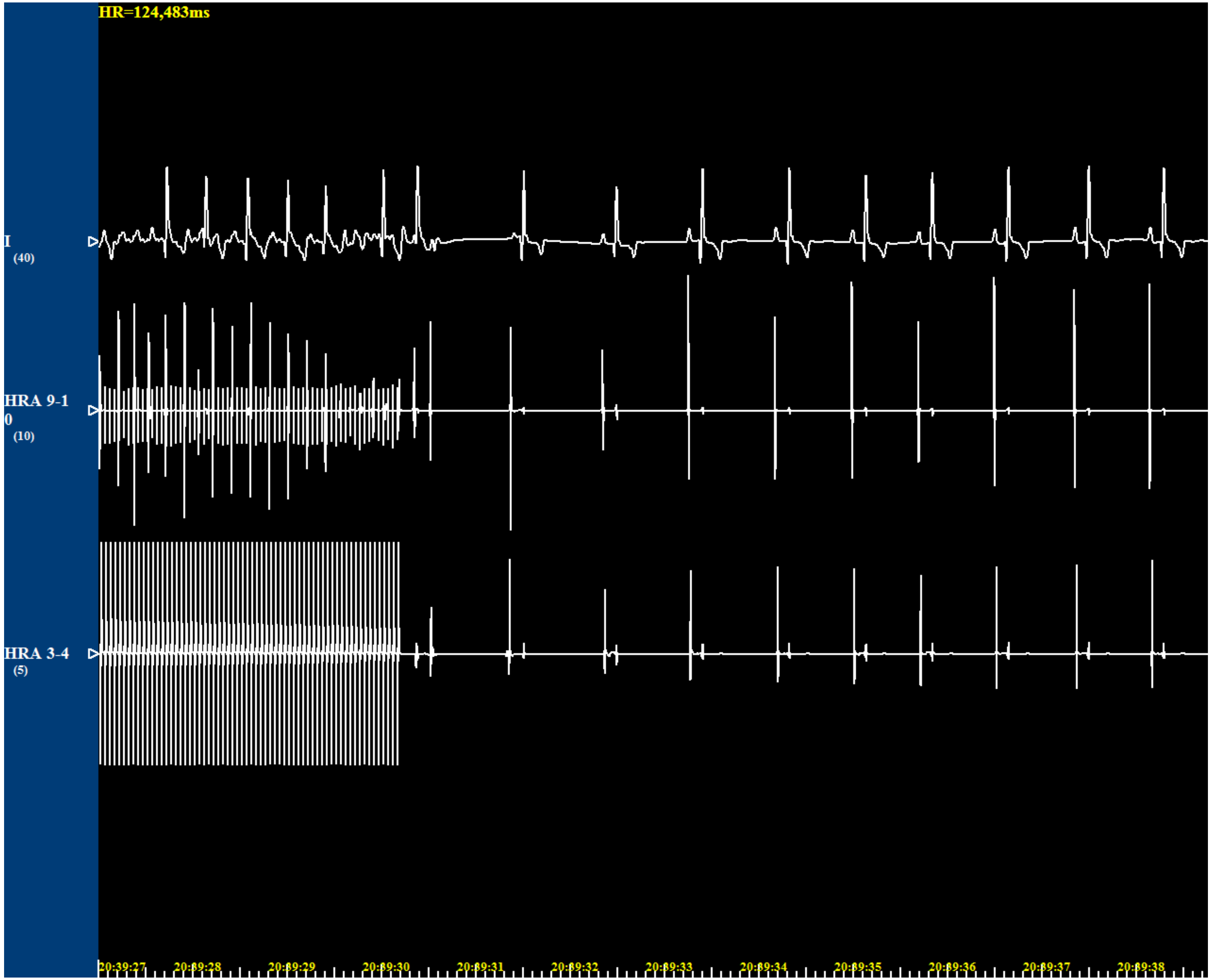
HR=124,483ms
I
(40)
HRA 9-1
0
(10)
HRA 3-4
(5)
20:39:27
20:39:28
20:39:29
20:39:30
20:39:31
20:39:32
20:39:33
20:39:34
20:39:35
20:39:36
20:39:37
20:39:38

C (Fig 2F)

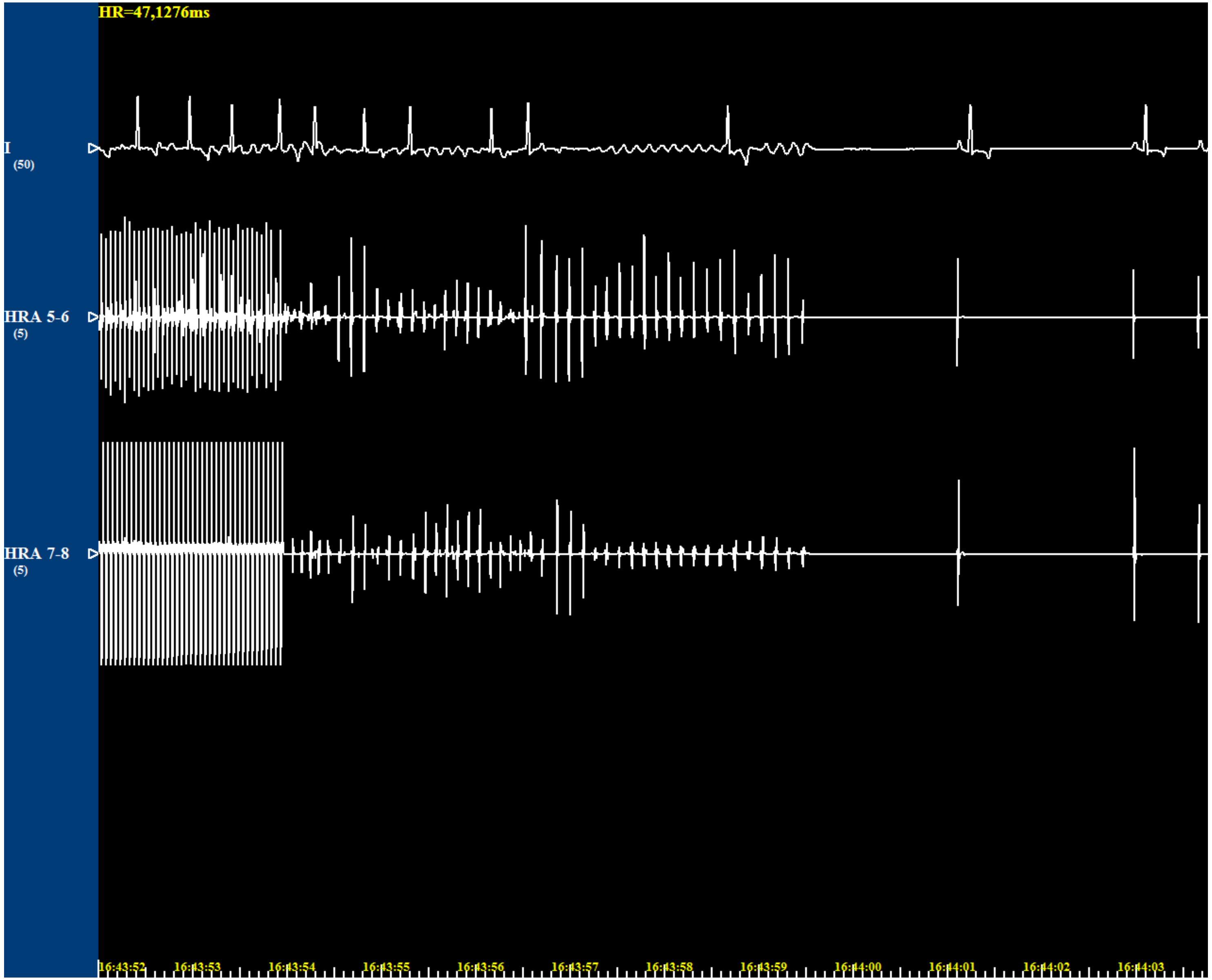
HR=47,1276ms
I
(50)
HRA 5-6
(5)
HRA 7-8
(5)
16:43:52
16:43:53
16:43:54
16:43:55
16:43:56
16:43:57
16:43:58
16:43:59
16:44:00
16:44:01
16:44:02
16:44:03

D (Fig 2G)

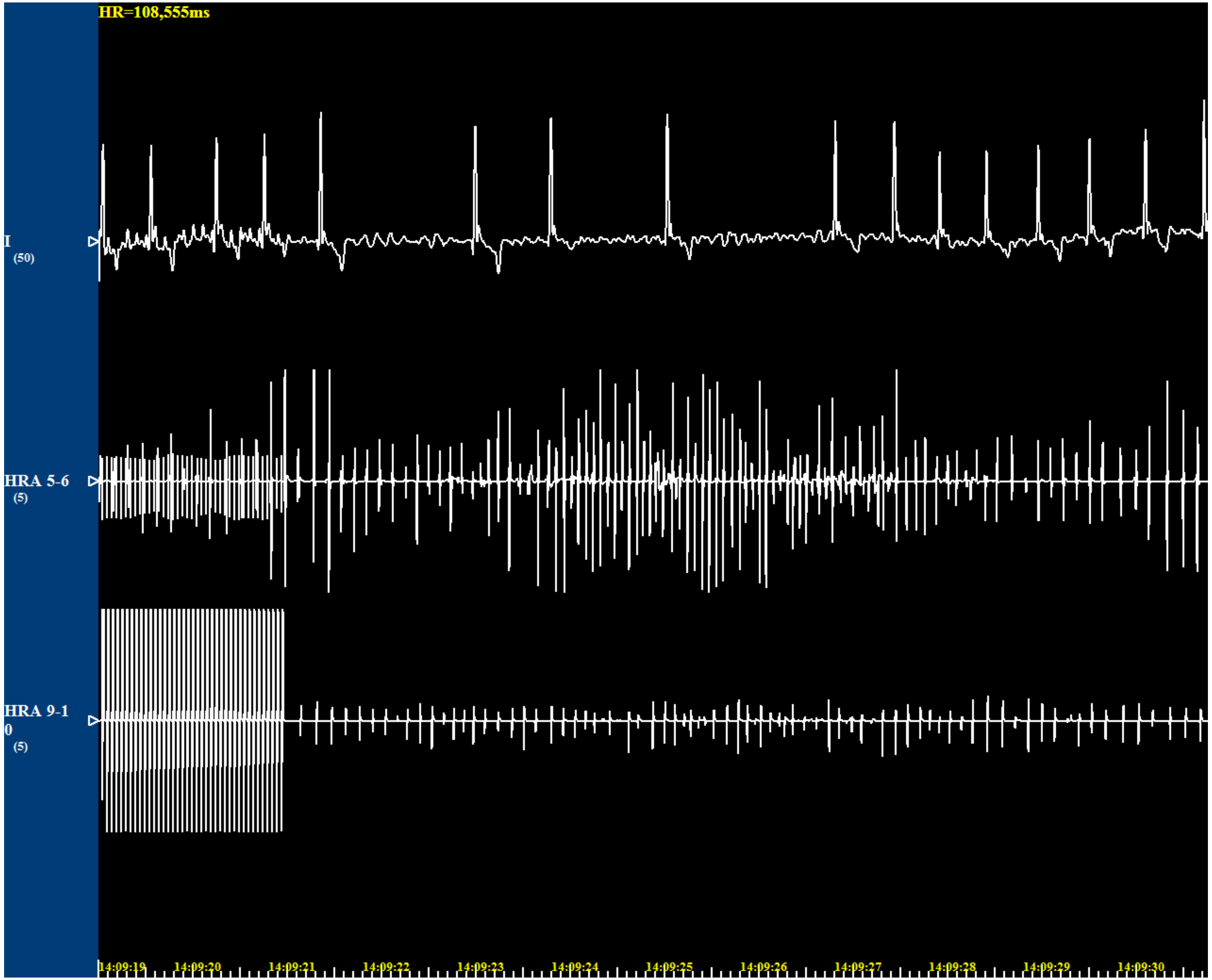
HR=108,555ms
I
(50)
HRA 5-6
(5)
HRA 9-10
(5)
14:09:19
14:09:20
14:09:21
14:09:22
14:09:23
14:09:24
14:09:25
14:09:26
14:09:27
14:09:28
14:09:29
14:09:30

E (Fig 2H)

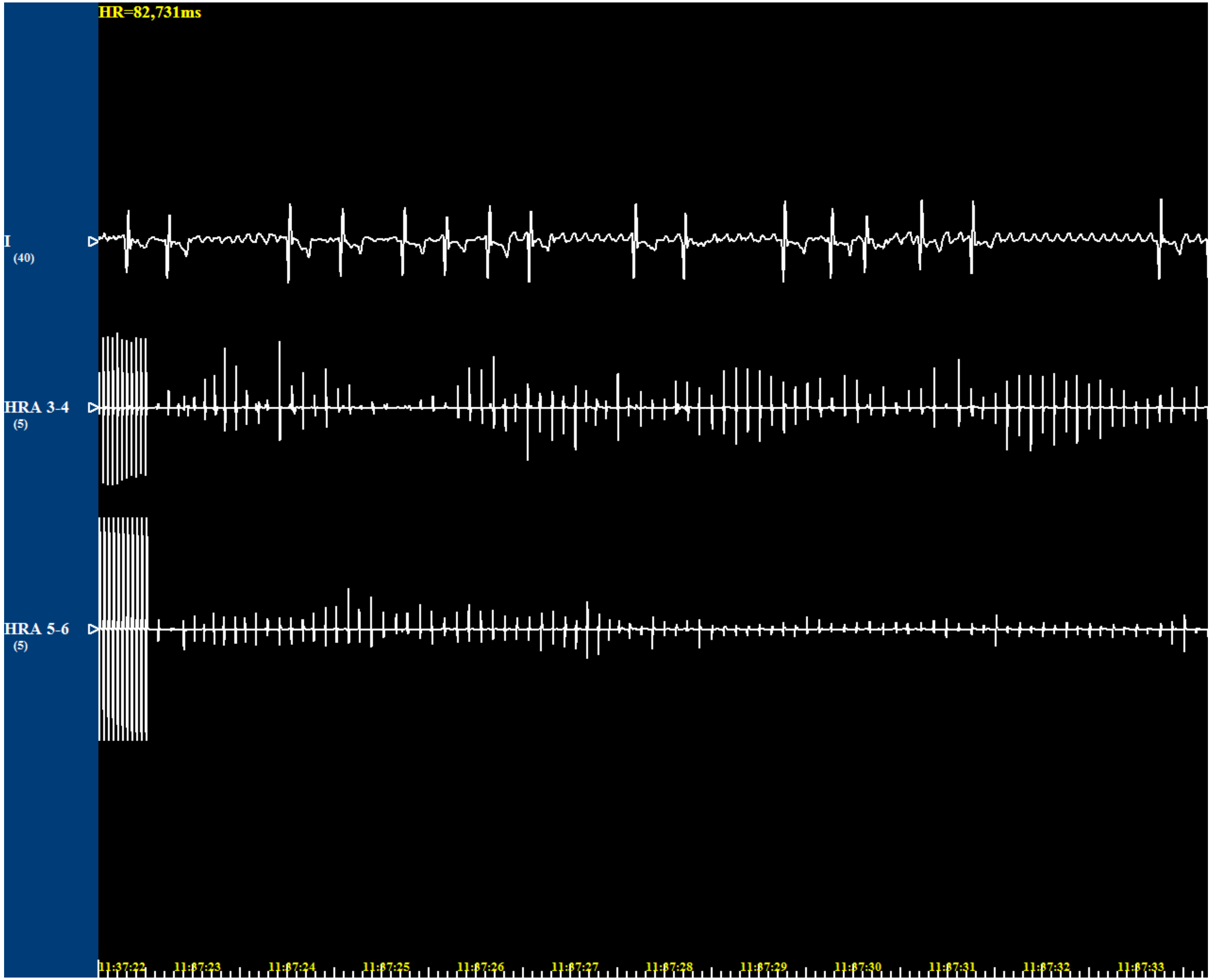

HR=82,731ms
I
(40)
HRA 3-4
(5)
HRA 5-6
(5)
11:37:22
11:37:23
11:37:24
11:37:25
11:37:26
11:37:27
11:37:28
11:37:29
11:37:30
11:37:31
11:37:32
11:37:33
